# Accounting for spatial variation in weather factors predicts spatial variations in *Culex quinquefasciatus* abundance in the Desert Southwest

**DOI:** 10.1186/s13071-026-07326-z

**Published:** 2026-03-11

**Authors:** Kayode Oshinubi, Crystal M. Hepp, Ye Chen, Eck Doerry, Nicole Busser, John Townsend, James Will, Irene Ruberto, Melissa Kretschmer, Joseph Mihaljevic

**Affiliations:** 1 School of Informatics, Computing, and Cyber Systems, Steve Sanghi College of Engineering, Northern Arizona University, Flagstaff, AZ USA; 2 Pathogen and Microbiome Division, Translational Genomics Research Institute, Flagstaff, AZ USA; 3 Department of Mathematics and Statistics, Northern Arizona University, Flagstaff, AZ USA; 4 Maricopa County Environmental Services Department, Vector Control Division, Phoenix, AZ USA; 5 Arizona Department of Health Services, Phoenix, AZ USA; 6 Maricopa County Department of Public Health, Phoenix, AZ USA

**Keywords:** West Nile virus, *Culex quinquefasciatus*, Clustering, Mosquitoes, Weather

## Abstract

**Background:**

Mosquitoes are vectors of diseases globally, making development of models that better explain mosquito abundance imperative. Mosquito population dynamics are particularly sensitive to local weather conditions, and mosquito-borne disease outbreaks can be spatially concentrated. There is a need for improved modeling studies to address whether spatial variation in disease outbreaks is driven by spatial variation in weather conditions, especially in dry and hot environments. In the present study, we built a climate-driven model of mosquito population dynamics and compared whether predictions of mosquito abundance at the county scale were improved by accounting for subcounty weather variation.

**Methods:**

Using a 5-year time series of weekly *Culex quinquefasciatus* abundance data collected for each zip code in Maricopa County, USA, we assessed how local variation in weather could explain and predict mosquito population dynamics. We built a mechanistic model of mosquito population dynamics influenced by daily maximum temperature and 30-day accumulated precipitation. We grouped zip codes on the basis of similar patterns of temperature and precipitation using functional clustering. We compared two approaches: one using county-level average weather and another using data from the identified weather clusters. We used Markov chain Monte Carlo simulations to fit the mechanistic model using averaged weather data in each cluster, then compared the model fit with observed data between the county-level model and the model on the basis of weather-based clusters.

**Results:**

Simple, weather-forced modeling accurately estimated detailed *Cx. quinquefasciatus* abundance trajectories throughout the 5-year period. Modeling mosquito abundances in the subcounty spatial clusters demonstrated that the same effects of temperature and precipitation on population growth rates could explain small-scale changes in mosquito abundances. However, when we aggregated the subcounty model fits to the county-scale, the resulting fits were more precise but sometimes overly confident, leading to lower overall accuracy and predictive performance.

**Conclusions:**

Our study demonstrated the importance of collecting fine-scale mosquito abundance data to improve our understanding and the predictability of mosquito population dynamics. The strong performance of both the cluster-based and county-level models illustrated the value of spatially sensitive modeling in this application. We anticipate that such modeling efforts will aid in using weather forecasts to predict increases in mosquito populations, thereby aiding in efforts to control the spread of infectious disease.

**Graphical abstract:**

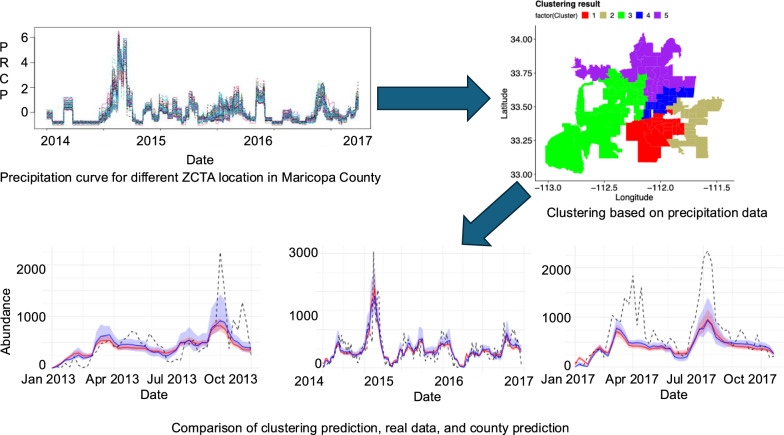

**Supplementary Information:**

The online version contains supplementary material available at 10.1186/s13071-026-07326-z.

## Background

Mosquitoes are carriers of many pathogens that have global impacts, including West Nile virus (WNV), yellow fever virus, Rift Valley virus, Zika virus, chikungunya virus, dengue viruses, and malaria parasites, which cause more than 700,000 deaths annually [1]. *Culex* are carriers of several encephalitis-causing pathogens that have significant public health impacts, including West Nile virus (WNV), St. Louis encephalitis virus (SLEV), Japanese encephalitis virus (JEV), and Western equine encephalitis virus (WEEV) [1]. WNV has become the leading cause of arboviral disease in North America since its introduction in 1999, with over 50,000 human cases reported in the USA [1], and epidemiological patterns driven by shifts in *Culex* feeding behavior [2]. The ecology of WNV transmission in North America involves complex interactions among climate, vectors, and avian hosts [3]. Furthermore, 1 in every 150 people infected with WNV is diagnosed with neuroinvasive WNV disease, with many suffering from long-lasting secondary sequelae (e.g., depression, memory loss, and motor dysfunction) [4–9].

*Culex* are ectotherms with complex life cycles and population dynamics linked to weather and local environmental conditions [4, 5], such as temperature, precipitation, humidity, and the availability of standing water for larval development. It is well documented from laboratory and field studies that temperature drives the rates of many key demographic traits across different *Culex* life stages [10–19]. Interestingly, humidity can alter the shapes of these thermal performance relationships, indicating that interactions between weather factors also impact population dynamics [7]. For *Culex* species, the availability of standing water for larval development helps explain trends in abundance, such that cumulative measures of precipitation are often used as proxies to model mosquito abundance [20–22]. A recent study [8] demonstrated that water availability strongly influences *Culex* abundance and WNV transmission across California, with the *Cx. pipiens* complex (which includes *Cx. quinquefasciatus*, the focus of our study) showing a 17-fold increase in abundance with irrigation activities in developed areas. While different *Culex* species exploit different breeding habitats—*Cx. tarsalis* in agricultural settings versus *Cx. quinquefasciatus* in urban environments—precipitation and water availability remain consistent drivers of population dynamics across species [8]. Accordingly, weather factors show statistical relationships with the burdens of many *Culex*-borne encephalitis viruses. For instance, statistical modeling studies often use weather factors to explain and predict the cases of WNV in humans [9–14]. On the basis of the relationships among climate, *Culex* mosquito population demography, and disease burden, more recent studies attempt to project the effects of climate change on the future global distributions of *Culex* and the diseases they transmit [23–28].

Given the complicated nature of climate’s effects on *Culex* population dynamics, flexible modeling approaches are needed to mechanistically explain how multiple climate variables influence variation in mosquito abundance patterns over space and time. *Culex* population dynamics can fluctuate at relatively small spatial scales, likely because local weather variation leads to spatial and temporal variation in mosquito abundances [20–22]. For instance, even across a few hundred square kilometers, *Culex* abundance can vary spatially and seasonally, potentially owing to differences in local precipitation and temperature, and the effects of weather may even vary among co-occurring *Culex* species [23, 26]. An important challenge is therefore not only to build mechanistic models that can explain temporal trends in *Culex* population fluctuations but that also interrogate the factors that explain spatial heterogeneities in these abundance patterns.

Many methods are used to model the spatial and temporal fluctuations in *Culex* population abundances, ranging from purely mathematical models (e.g., compartmental differential equation models) to purely statistical models (e.g., linear regression frameworks). Developing an adequate mathematical or statistical model to explain or predict *Culex* population dynamics in relation to climate is a challenging endeavor with various trade-offs. Purely statistical models, including statistically based machine learning algorithms, may explain temporal and spatial trends in *Culex* abundances [8, 9, 24–27]. A challenge with statistical modeling approaches is that they typically are inadequate representations of the well-known nonlinear effects of climate on complex population dynamics. Additionally, statistical models are generally correlational, meaning that at least some mechanistic understanding of the system is limited. Fully mechanistic [29–31] and contemporary mathematical models often introduce large sets of differential equations that represent the various life stages of mosquitoes, from egg to larvae to adult, and may include the effects of temperature on certain demographic rates [32–36]. Such detailed models have shown considerable success in predicting mosquito population dynamics for species such as *Aedes aegypti* when extensive laboratory and field data are available to parameterize stage-specific processes [37]. Such models not only have the strong benefit of inherently including nonlinear effects of climate on demographic patterns, but they also have some challenges. It is difficult to derive realistic estimates for the many parameters in such large models (i.e., a dimensionality problem) when working with typical surveillance data that only measure adult abundance. Parameterizing such models therefore often relies on laboratory experiments that measure thermal performance of mosquitoes, but such parameter estimates are largely untested in field settings (although see [38]). Fortunately, recent mechanistic modeling and model-fitting approaches show promise in incorporating both temperature and precipitation to explain dynamic *Culex* population abundance patterns [15, 36].

In our study, we sought to develop a semi-mechanistic modeling approach that balances mechanistic representation of population processes with flexible statistical characterization of climate effects. This approach was designed to be tractable for typical surveillance datasets that measure only adult mosquito abundance, while maintaining sufficient mechanistic structure to capture known nonlinear relationships between climate and mosquito population dynamics. This modeling approach allowed us to explore whether spatiotemporal variation in weather explains the nonlinear dynamics of *Culex* populations in natural landscapes. We tested our modeling approach using *Cx. quinquefasciatus* in Maricopa County, AZ, which transmits multiple infections to humans, and is the most abundant vector for West Nile and St. Louis encephalitis (SLEV) viruses in the county. According to the Centers for Disease Control and Prevention (CDC)^1^, Maricopa County consistently is among the top ten counties in the nation for yearly West Nile virus cases [1]. From 1999 to 2022, Maricopa County had the most human cases in the nation, with a total of 3006 case reports. Hundreds of traps are set weekly, and mosquito abundances can vary dramatically in magnitude across subdivisions of the county. Hence, this system was ideal for testing how a combination of weather factors may influence the spatiotemporal heterogeneity in *Culex* population dynamics. We therefore hypothesized that accounting for microweather variation—by grouping areas with similar weather patterns—would improve our model’s ability to predict mosquito abundance across the county. This modeling study was important because it elucidated how spatial variation in weather affects spatial variation in *Culex* abundance. Moreover, in future work, the simplified modeling approach we developed here could be incorporated into epidemiological models to account for important weather and spatial variation in *Culex*-borne encephalitis virus human case reports.

## Methods

### Approach overview

First, we developed a semi-mechanistic model of the temporal dynamics of adult mosquito abundance, which incorporated nonlinear statistical effects of temperature and accumulated precipitation. We defined “semi-mechanistic” as an approach that mechanistically represents population growth processes while using flexible statistical functions to characterize climate effects on demographic rates. This differs from fully mechanistic stage-structured models that explicitly simulate each life stage (egg, larva, pupa, adult) with temperature-dependent developmental rates and stage-specific parameters derived from laboratory experiments (e.g., Romeo Aznar et al. [37]). Although such detailed models have successfully predicted mosquito dynamics for species such as *Ae. aegypti* at small spatial scales, they require extensive parameterization of stage-specific demographic processes. Our approach prioritized fitting temporal patterns in field-collected trap data (which measure only adult abundance) while maintaining key mechanistic structure, making it more tractable for large-scale surveillance datasets and operational public health applications. We then used Bayesian Markov chain Monte Carlo (MCMC) to fit our model to 3 years of weekly, adult female *Cx. quinquefasciatus* abundance observations across Maricopa County (i.e., the abundance data aggregated across all encephalitis vector surveillance (EVS) traps baited with ${\mathrm{CO}}_{2}$ [39–42] established in the county). To test whether spatial heterogeneity in weather explains variation in the patterns of *Cx. quinquefasciatus* abundance across Maricopa County, we clustered ZIP codes by local similarities in weather and aggregated the mosquito abundance across traps within these clusters. We developed a method to hierarchically re-fit our model to abundance data in each cluster. Next, we compared whether fitting the data per cluster and then aggregating these fits to the county level improved the overall fit of the county-level data. In this way, we assessed whether heterogeneity in weather-based spatial clusters helped better explain the county-level aggregated data. Finally, we also evaluated the out-of-sample prediction accuracy of our models, at both county and cluster scales. Below, we describe each step of the process in detail.

### Climate-forced mathematical model

We developed a mathematical model that explains temporal patterns of female mosquito abundance, which is driven by two exogenous climatic variables: temperature and precipitation. The general form of the ordinary differential equation is

$$\frac{dX(t)}{dt}=\nu f({\mathrm{Temp}}(t),{\mathrm{Prcp}}(t))-\mu X(t).$$

$X(t)$ is female *Cx. quinquefasciatus* abundance over time, with model time steps discretized to daily as $X({t}_{i})$ where $i=1,2,3,\dots$. The model assumed that there is a baseline population growth rate of mosquitoes, $\nu$, which is modulated by a statistical function of daily measures of temperature ${\mathrm{Temp}}(t)$ and precipitation ${\mathrm{Prcp}}(t)$. Specifically, ${\mathrm{Temp}}(t)$ is the daily maximum temperature in degrees Celsius, and ${\mathrm{Prcp}}(t)$ is the daily measure of 30-day accumulated millimeters. We scaled the precipitation variable by dividing by 50 to improve numerical stability during MCMC parameter estimation. This scaling factor was chosen on the basis of the observed range of 30-day accumulated precipitation in Maricopa County, which ranged from 0 to approximately 230 mm during the study period (with typical non-monsoon values of 0–50 mm and monsoon season peaks of 100–230 mm). Dividing by 50 places the scaled precipitation variable on a 0–5 range, which is comparable in magnitude to the temperature effects and facilitates stable parameter estimation. Note that the choice of scaling constant (50) does not affect model fit or biological interpretation, as it is absorbed into the estimated parameters; rescaling the precipitation variable simply results in proportionally rescaled parameter estimates with identical model predictions.

The form of the statistical function is:

$$f({\mathrm{Temp}}(t),{\mathrm{Prcp}}(t))=-({\mathrm{Temp}}(t)-Tmin)({\mathrm{Temp}}(t)-Tmax))\frac{1}{(1+{\mathrm{e}}^{\alpha -\phi {\mathrm{Prcp}}(t)})}$$

The temperature effect is modeled as a quadratic equation, representing a thermal performance curve of population growth rate, controlled by a minimum $Tmin$ and a maximum $Tmax$ growth temperature. This structure is agnostic to the fact that many traits across life stages have unique thermal performance curves [6]. Our strategy is instead to test a simplification by estimating a parsimonious representation of thermal effects on the overall growth rate of the adult mosquito population, integrating across the thermal effects of specific, life-stage-specific traits.

The precipitation effect is modeled as a logistic, saturating function, whose shape is controlled by constants $\alpha$ and $\phi$. Biologically, this means that as accumulated precipitation increases, the growth rate increases, but the model could estimate how quickly this increase occurs across the range of precipitation and whether there is a saturating effect, above which more accumulated precipitation has negligible effects. Illustrative plots of the temperature and precipitation functions and their corresponding seasonal time series are shown in Additional File 1: Supplementary Fig. S1.

We fixed the per‑capita mortality rate $\mu$ = 0.12 (mean adult mosquito lifespan ≈8.3 days), consistent with field estimates for *Culex* mosquitoes [43–45], instead of estimating it. Because the baseline growth rate ($\nu$) and mortality rate ($\mu$) are not independently identifiable from abundance data alone—only their difference (the net growth rate) determines observable population dynamics—we fixed $\mu$ at a biologically plausible value to enable estimation of $\nu$. Estimating both would create an identifiability issue. Alternative combinations of $\mu$ and $\nu$ producing the same net growth rate would yield equivalent model fits, meaning the specific value of $\mu$ does not affect model interpretation. Consequently, fluctuations in the fitted growth term implicitly capture both births and deaths.

In Table 1, we described each parameter in our mathematical model.

**Table 1:** Parameters of the model

Parameter	Signification of parameters	Unit	Source
*Ν*	Baseline population growth rate	$\frac{\mathrm{mosquitoes}}{{}^{\circ }C\cdot {\mathrm{day}}}$	Estimated
*T*_min_	Minimum temperature that constrains population growth	${}^{\circ }C$	Estimated
*T*_max_	Maximum temperature that constrains population growth	${}^{\circ }C$	Estimated
*Α*	Contributes to the inflection point of the precipitation effect curve	Unitless	Estimated
*Φ*	Steepness of the precipitation effect curve	1/mm	Estimated
*µ*	Mosquito death rate	1/day	Fixed

### Mosquito abundance data

**Fig. 1:**
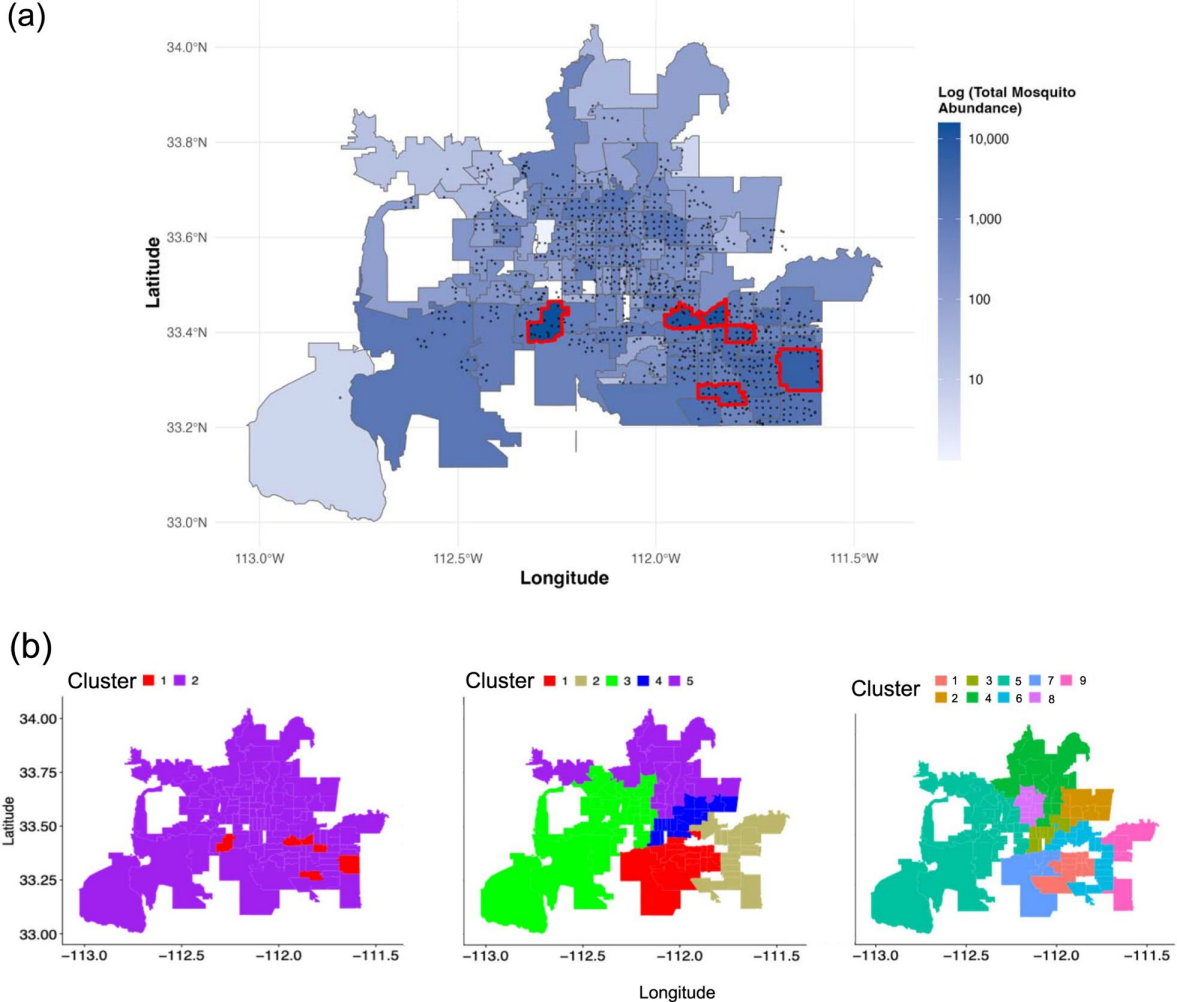
**a** Spatial distribution of total *Culex quinquefasciatus* female abundance in Maricopa County, Arizona (total area ~23,890 km^2^). The choropleth map shows cumulative mosquito abundance by ZIP Code Tabulation Area (ZCTA, average area ~219 km^2^) on a log10 scale, with darker blue indicating higher abundance. Black points represent CO_2_-baited trap locations (*n* ≈ 800) monitored weekly by the Maricopa County Vector Control Division. Red boundaries highlight six outlier ZCTAs with exceptionally high abundance. Total abundance ranges from 1 to 15,632 mosquitoes per ZCTA over the 3-year period (2014–2016), with highest concentrations in central urban areas. **b** Clustering result; two clusters represent six outlier ZCTAs (red) versus all others (purple), whereas the five and nine clusters are clustered on the basis of precipitation data of all other ZCTAs

The mosquito abundance data used in this study were collected by the Maricopa County Environmental Services Vector Control Division [42]. Mosquitoes were collected using encephalitis vector surveillance (EVS) traps (BioQuip Products, Rancho Dominguez, CA, USA) baited with dry ice (approximately 1–2 kg per trap) as a CO_2_ attractant [39, 40]. Data were collected weekly from approximately $800$ ${\mathrm{CO}}_{2}$ traps distributed throughout Maricopa County, with trap placement concentrated in populated and accessible areas (Fig. 1a). Total mosquito abundance varied substantially across the county, with cumulative abundance over the 2014–2016 period ranging from 1 to 15,632 *Cx. quinquefasciatus* females per ZIP Code Tabulation Area (ZCTA) (median = 501, mean = 1088), demonstrating considerable spatial heterogeneity even at relatively small spatial scales (Fig. 1a). The longitude and latitude of trap locations were documented for spatial analysis. Each trap was placed for a 12-h collection period once per week for 50 weeks of the year. Collections were subsequently sorted by mosquito species and sex. Data used in this study were restricted to *Cx. quiquefasciatus* females, the most frequently trapped mosquito vector in Maricopa County, which is known to transmit avian malaria, WNV, SLEV, and other pathogens. This species constitutes approximately 80% of pathogen-positive mosquito pools in Maricopa County, making it the primary vector of concern for these encephalitis viruses.

In our analysis, we used a training data set spanning 3 years, from 1 January 2014 to 31 December 2016, and we used 2013 and 2017 data to validate our model as an out-of-sample prediction data set. We chose 2014–2016 for the training set, because the data include unique patterns in abundance that can help validate the effects of weather variation. For instance, in 2014, there was a large monsoon season, and this effect can be visually observed in the abundance data, while years 2015 and 2016 had relatively average abundance trends. For the county-level data set, weekly mosquito abundance was aggregated as the total number of adult female *Cx. quinquefasciatus* captured across all routine (RT) CO_2_ traps operating that week (i.e., summed across all traps). We used data for adult female mosquitoes only because they were host-seeking and attracted to ${\mathrm{CO}}_{2}$ traps. We did not standardize by the number of traps because our focus was on temporal dynamics and relative changes in abundance patterns rather than absolute density; the number of routine traps remained relatively stable throughout the study period (~800 traps county-wide). Each trap is identified by GPS coordinates (longitude and latitude), such that for the cluster-level data sets, we first aggregated trap data to the ZIP code-level (i.e., ZIP Code Tabulation Areas, ZCTA) by summing weekly female counts across all routine traps within each ZCTA, and then clustered ZCTA by weather similarity and aggregated trap data accordingly (Fig. 1a).

### Temperature and precipitation data

**Fig. 2:**
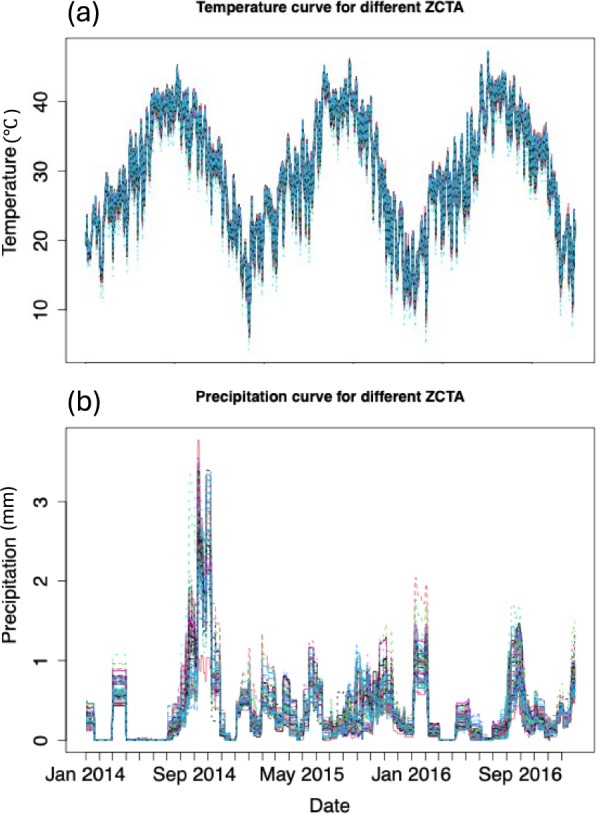
Daily climate variables in Maricopa County from 2014 to 2016. **a** Daily maximum temperature (°C) and **b** daily precipitation (mm), both averaged across all ZCTAs within Maricopa County using 4 km × 4 km PRISM gridded data. These aggregated time series were used as climate inputs for both county- and cluster-level mosquito abundance models. The different colors represent temperature and precipitation for each ZCTA

Daily estimates of maximum temperature in Celsius and precipitation in millimeters were downloaded from PRISM via the prism R package [46]. We downloaded the Prism daily data in a 4 km × 4 km raster. For the county-level model, we averaged daily values across all the Prism data in the raster within the Maricopa County polygon defined by the 2010 Census Bureau delineations (tigris package [47]) in Maricopa County. For the cluster-level model, we averaged the Prism data across the grid within each ZCTA polygon, again using 2010 Census Bureau delineations. Then, we averaged these data across all ZCTAs included in a given cluster. In the model, we used 30-day accumulated precipitation, scaled by dividing by the constant 50. In Fig. 2, we present the time-series plot for daily maximum temperature and 30-day accumulated precipitation for 2014–2016, which is averaged across ZCTA in Maricopa County.

We explored the spatial distribution of the mosquito abundance data present within the 109 non-outlier ZCTAs across Maricopa County (see details of outlier ZCTAs in Additional File 1: Supplementary Text S1). Our goal was to cluster these ZCTAs on the basis of similarities in climatic data during the 3-year window of the training data set. We used only the precipitation time-series data, as the temperature time-series did not vary significantly across the county (Fig. 2). Formal functional principal component analysis revealed that temperature explained > 98% of spatial variance across ZCTAs (PC1), whereas precipitation showed substantial spatial heterogeneity with variance distributed across multiple principal components (Additional File 1: Supplementary Fig. S5). Temperature-based clustering produced unstable results with nearly indistinguishable mean temperatures per cluster and varying cluster assignments across runs (Additional File 1: Supplementary Fig. S6). In contrast, precipitation-based clustering yielded stable, geographically coherent groups. When both variables were combined in functional clustering (FunFEM) [48], results were identical to precipitation-only clustering, demonstrating that temperature provided no additional clustering information. Detailed analyses are provided in Additional File 1: Supplementary Text S2. Quantitative assessment revealed that the coefficient of variation (CV = spatial standard deviation [SD]/mean) for temperature across ZCTAs was only 0.021 (mean spatial SD = 0.64 °C), compared with 0.30 for precipitation (mean spatial SD = 0.11 mm)—a 14-fold difference in relative spatial heterogeneity (see Additional File 1: Supplementary Figs. S7, S8 and Supplementary Text S2 for detailed analysis). Using the precipitation data, we clustered the ZCTAs on the basis of a functional time-series clustering technique implemented in the FunFEM package in R [48]. This clustering technique requires the user to specify the number of clusters desired. Therefore, we tested five and ten clusters. Briefly, to apply functional time-series clustering, first, a basis function must be fit to the time-series data to smooth it. We used a Fourier basis, which is commonly applied for seasonal environmental data [49]. Although Fourier bases are typically most appropriate for strictly periodic patterns, they can also capture broader seasonal trends in episodic data such as precipitation. As shown in Fig. 2b, precipitation in Maricopa County was episodic and event-driven rather than smoothly periodic, with irregular monsoon events creating spikes in accumulation. The Fourier basis smoothed these irregular patterns to capture the underlying seasonal structure (higher accumulation during monsoon season, lower during winter). This level of smoothing was appropriate for our clustering objective, which was to identify ZCTAs with similar seasonal patterns of precipitation timing and magnitude rather than to precisely replicate individual storm events.

The clustering algorithm compared time-series data by transforming them into functional forms, often using basis expansions such as splines or Fourier series, to capture their continuous-time dynamics [49]. It then measured similarity on the basis of shared features such as trends, seasonal patterns, or amplitude variations using a model-based clustering approach in a discriminative functional subspace, where differences between curves were captured via latent variable modeling and Gaussian mixture models, primarily relying on functional principal component analysis (PCA) and Fisher-like discriminative analysis. Time series with similar functional properties were grouped into contiguous clusters, allowing for the identification of common behaviors and trends across the data while accounting for variability in shape, scale, and temporal alignment.

Note that when we specified ten clusters, one cluster only included four ZCTAs, each with a maximum mosquito abundance per week of less than ten. Therefore, we manually moved those to the most similar other cluster, leaving us with a comparison of five versus nine clusters (see middle and last panel of Fig. 1b).

### Fitting the model to the time-series data

We used a Metropolis–Hastings Markov chain Monte Carlo (MCMC) algorithm to sample from the joint posterior distribution of model parameters. To calculate the likelihood, we compared the solution of our ordinary differential equation model with the weekly observed abundance data. We solved the differential equation model with the deSolve [50] package using the method lsoda [51] in R, incorporating daily fluctuations in temperature and precipitation. We compared the model with the data every 7 days; therefore, although the model ingested daily data on weather, the model was only compared with the observed mosquito data once per week. The likelihood was defined as a negative binomial probability distribution with an estimated over-dispersion parameter (see Additional File 1: Supplementary Texts S3, S4 for more details). For each spatial data set, we ran four MCMC chains, each with 5000 iterations, in parallel using the futures R package [52]. We used the $\widehat{R}$ statistic [53], also known as the Gelman-Rubin diagnostic, to assess the convergence of the chains. We also performed posterior predictive checks, described below, to ensure the joint posterior defined a reasonable parameter estimation space.

We fit the model to the following five data partitions: county-level with outliers, county-level without outliers, two-cluster (outlier ZCTA versus all other ZCTAs), five-cluster without outliers, and nine-cluster without outliers. For the two county-level partitions, we ran a standard MCMC process. For the cluster-level partitions, we ran a hierarchical inference approach.

### Model validation

We used within-sample and out-of-sample predictive measures to compare model performance across the different data partitions. We were ultimately interested in understanding whether accounting for spatial variation in weather and baseline growth rates might improve the model’s explanation of county-wide, total mosquito abundances. Therefore, in calculating goodness-of-fit metrics, for the cluster-level data partitions, we summed metrics across clusters to a county-level performance metric.

The method we employed for goodness-of-fit measurement was root mean square error (RMSE). To generate this measurement for each data partition and model fit, we first generated posterior predictive model runs. For each data partition and MCMC analysis, we randomly sampled 100 parameter sets from the joint posterior. For each parameter set, we solved the ordinary differential equation (ODE) numerically at daily time steps. Because mosquito abundance data were collected weekly, we sampled the daily model output at weekly intervals (i.e., at the end of each 7-day collection period) to match the temporal resolution of the observations. Across the 100 posterior predictive model runs, we calculated the median model prediction for each week. The RMSE was then calculated by comparing the observed weekly mosquito abundance with the median weekly model prediction, summing the squared errors across all weekly observations and, when appropriate, across clusters.

## Results

### Variation in precipitation and temperature

**Fig. 3:**
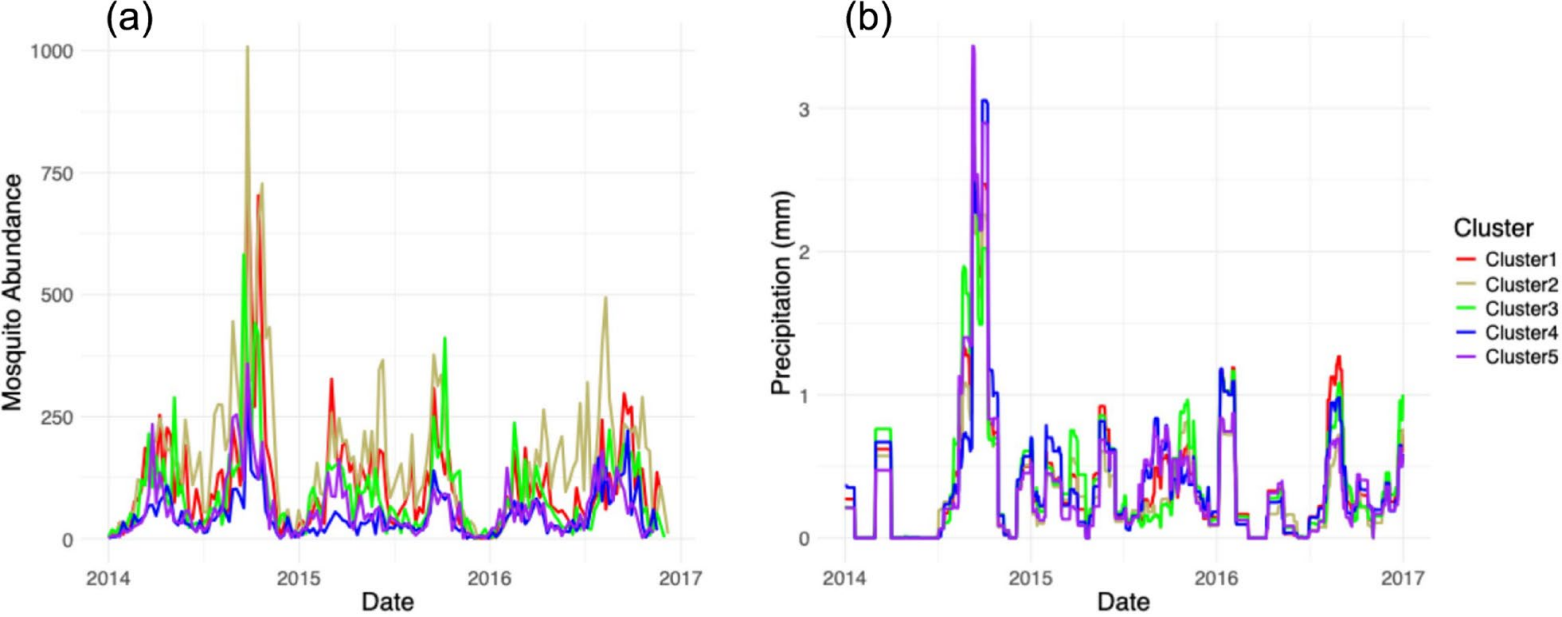
Spatiotemporal patterns in precipitation and mosquito abundance across five climate-based clusters of ZCTAs in Maricopa County. **a** Weekly mosquito abundance, highlighting cluster-level variation in response to climatic conditions, particularly during the 2014 monsoon season and mid-to-late 2015. **b** 30-day accumulated precipitation (mm), showing differences in precipitation magnitude and timing among clusters

The clustering routine captured logical spatial groupings of ZIP codes (ZCTA) across Maricopa County on the basis of spatial and temporal variation in 30-day accumulated precipitation (Figs. 2, 3a,b). When we examined the PRISM-derived precipitation data [54], we observed that clusters were primarily differentiated by precipitation magnitude rather than timing. For instance, in the five-cluster case, all clusters experienced precipitation during the same monsoon events in 2014 and 2015, but with varying total accumulation (Fig. 3a,b). The high degree of temporal synchrony in precipitation across clusters indicated that most precipitation events occurred at the county scale rather than as highly localized events. This pattern may reflect both the actual spatial scale of monsoon storms in the region and limitations of gridded climate products for capturing fine-scale spatial heterogeneity. PRISM data (4 km resolution) are derived from spatial interpolation of weather station data, which can smooth out small-scale variability in localized convective precipitation events [55, 56]. Such fine-scale heterogeneity could potentially create more spatially variable mosquito breeding habitat availability than is captured by our clustering approach. Despite this limitation, the magnitude differences in precipitation across clusters were sufficient to produce detectable differences in baseline mosquito abundance patterns, indicating that even county-scale variation in precipitation accumulation affects mosquito population dynamics.

### Comparing county- and cluster-level modeling

**Fig. 4:**
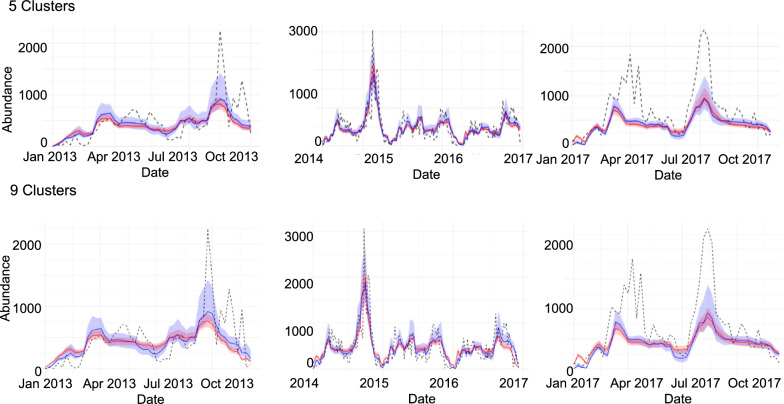
Comparison of clustering prediction, real data, and county prediction for five (top) and nine (bottom) clusters. Shaded ribbons in blue indicate 95% credible intervals for county prediction, while shaded ribbons in red indicate 95% credible intervals for cluster sum prediction. The dotted black lines represent observed mosquito abundance data over time

**Fig. 5:**
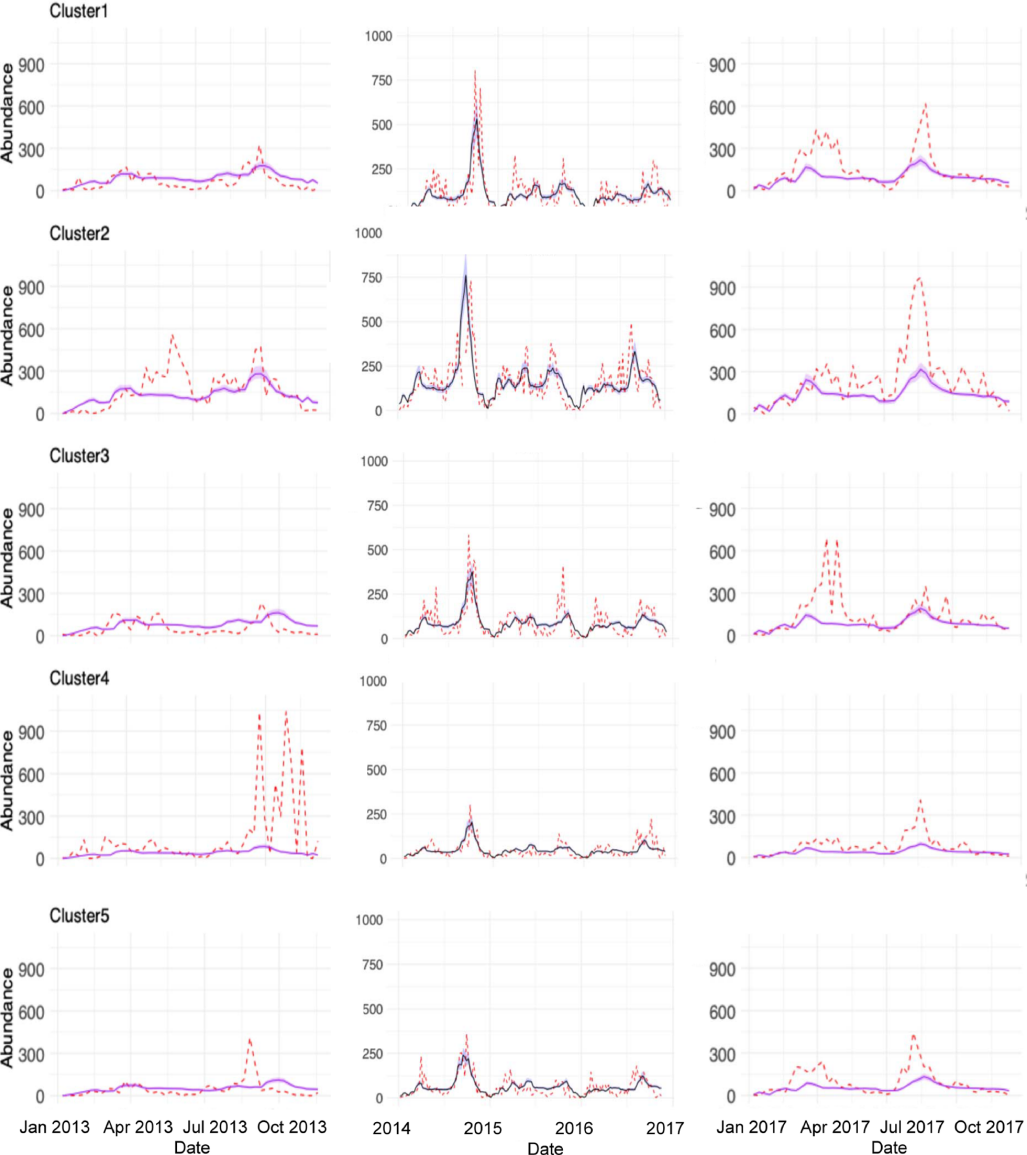
Five-cluster prediction. Shaded ribbons indicate 95% credible intervals from the county fit. Dotted red lines are the real data, while the black line is the model fit for within-sample fits, and the orange line is for out-of-sample fits

We aggregated cluster-level predictions to the county scale by summing predicted abundances across clusters (using an unweighted sum to parallel how observed trap data are aggregated), allowing direct comparison with county-level model fits. In general, our mechanistic model captured detailed patterns of mosquito abundance changing through time across multiple spatial scales for the 3 years of training data, particularly when we remove the six outlier ZCTAs (Figs. 4, 5 and Additional File 1: Supplementary Fig. S9). For example, the model generally captured the effects of high summer temperatures, where we saw corresponding declines in mosquito abundances, and the effects of monsoonal activity, where we can see dramatic increases in mosquito abundances due to heavy rainfall (e.g., in the second half of 2014). Although our model does not explicitly represent behavioral estivation, the temperature-dependent reduction in population growth during hot, dry periods followed by rapid increases after monsoon rains may functionally capture similar dynamics, whether driven by demographic processes or behavioral dormancy.

Accounting for heterogeneity in precipitation across clustered ZCTAs led to higher precision in cluster-level model fits, as evidenced by narrower credible intervals. However, when we aggregated these cluster-level predictions to the county scale, the resulting fits were sometimes overly confident, such that the credible intervals did not always capture the true observations (Fig. 4).

**Fig. 6:**
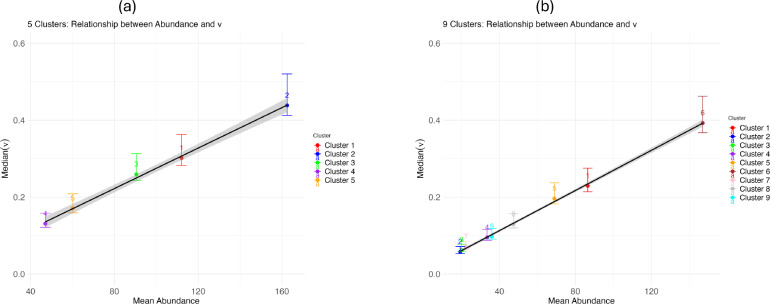
Association between mean mosquito abundance and baseline growth rate *ν* for **a** five-cluster and **b** nine-cluster spatial configurations. Points show median *ν* estimates with 95% credible intervals for each cluster. Linear regression (black line with 95% confidence bands) demonstrates a significant positive relationship between mosquito abundance and *ν* in both clustering schemes

Therefore, the county-level model, which used daily values for temperature and precipitation that were averaged across the ZCTA, had a relatively better fit compared with the aggregated cluster-level models, based on the root mean squared error (RMSE) metric (Table 2). Furthermore, there were no clear differences in the aggregated county-level model fit when we compared the five-cluster with the nine-cluster situations (Fig. 4). When we examined model fits per cluster, however, we did see that the model captures variation in mosquito abundances across clusters, explained mostly by differences in baseline population growth rates (Figs. 5, 6 and Additional File 1: Supplementary Fig. S9). Additionally, we conducted a regression analysis demonstrating the relationship between mean mosquito abundance and *ν* across clusters. This analysis showed a significant positive relationship (five clusters: *R*^2^ = 0.9969, *P* = 4.737 × 10^−5^; nine clusters: *R*^2^ = 0.9984, *P* = 3.266 × 10^−11^), providing quantitative evidence that variation in *ν* strongly predicts observed abundance differences across spatial clusters. In addition, we observed that the RMSE values vary across clusters depending on the number of clusters (Additional File 1: Supplementary Tables S3, S4). This result demonstrates that our method of hierarchical estimation of cluster-specific growth rates successfully characterized key differences among clusters. Collectively, the results indicated that the aggregate, county-level patterns in mosquito abundance can be most parsimoniously explained by a spatial average of temperature and precipitation data. Yet, the cluster-level models provided accurate, finer-scale inference of how a mosquito population varies across space and time, though at this finer scale, the model fits may be more prone to small prediction errors. Additional details about outliers and county-level analysis can be found in Additional File 1: Supplementary Text S5, S6 (Table 2).

**Table 2:** Model evaluation comparison at the county level

Model	Fitted RMSE (2014–2016)	Predicted RMSE (2013)	Predicted RMSE (2017)
County without “outlier” ZCTAs	252.6900	306.0828	494.7659
Five-cluster sum	265.8615	325.5769	507.0085
Nine-cluster sum	269.4774	349.3141	511.3981

### Out-of-sample performance

When we predicted mosquito abundance for two additional years of withheld data (2013 and 2017), our model still captured the fundamental characteristics of these mosquito abundance time series (see Fig. 5 [first and last panel] and Additional File 1: Supplementary Figs. S10, S11). Indeed, the key results from the training data still held true with the out-of-sample data. Particularly, the county-level model performed nearly equally as well as the cluster-level models. Notably, however, across spatial scales, the dynamic model did a poor job at explaining the early year increases in mosquito abundance observed in 2017, and underpredicted peaks in abundances observed in the later half of both 2013 and 2017. We observed these failures of the model in both the county- and cluster-level out-of-sample model fits.

## Discussion

Our research has shed light on the complex relationships among climate, spatial heterogeneities, and mosquito populations. By implementing a novel mechanistic model with a data-driven clustering strategy, we showed that a relatively simple mathematical model that incorporates temperature and precipitation effectively captures the dynamical patterns of mosquito abundance observed over multiple years in a large urban setting. When we fit our model to aggregate data for all of Maricopa County, our climate-driven model accurately explained the seasonal increase in mosquito abundances in the spring, a decline in the hottest part of the summer, and another increase in the warm, wet monsoon season. When we subdivided the county into clusters on the basis of similar weather patterns, we observed that average mosquito abundances varied substantially across the county. However, the cluster-level model still explained a substantial proportion of variance in these downscaled data, indicating that mosquito populations across the region exhibit consistent numerical responses to temperature and precipitation. When we aggregated these smaller-scale model fits, we improved precision around the median model prediction, but we did not improve county-level model accuracy—likely due to error accumulation across clusters and the sparse data within each group. What this tells us is that the same climate-forced model can be applied at subcounty spatial scales and will explain the key patterns of mosquito population dynamics; yet, at these smaller spatial scales, the model will be more prone to observational error, suggesting a trade-off between spatial resolution and model accuracy. Our results underscore the importance of modeling spatial structure when interpreting mosquito dynamics, particularly in regions such as the Desert Southwest where precipitation and temperature vary dramatically over small spatial scales. This variation across urban landscapes can be attributed to factors such as persistent high-pressure systems (heat domes) that amplify urban heat island effects, trapping extreme heat and creating pronounced temperature gradients between urban cores and surrounding areas [57–60]. Additionally, Arizona’s bimodal precipitation regime—characterized by two primary rainy seasons: winter frontal systems (typically October–March) and the summer North American Monsoon (July–September)—contributes to heterogeneous moisture patterns, with the monsoon often accounting for a substantial portion (frequently over 50%) of annual rainfall in the state [61, 62]. In 2014, an exceptionally active monsoon season, influenced by tropical moisture from the remnants of Hurricane Norbert, resulted in record-breaking precipitation, including a single-day total of 3.30 in. at Phoenix Sky Harbor Airport on 8 September—the highest calendar-day rainfall on record since 1895—and leading to intense, localized flooding and uneven hydrological impacts across the urban gradient [63, 64].

Previous studies have demonstrated that local weather conditions influence mosquito abundance and distribution [15, 20, 65, 66]. Research on *Cx. quinquefasciatus*, the focal species in our study, has highlighted how temperature and precipitation shape its life cycle. For example, a study in mountainous Hawaii [67] found that *Cx. quinquefasciatus* abundance increased with temperature, peaking at mid-elevation sites, and showed a nonlinear relationship with precipitation—highlighting temperature as a key driver of mosquito distribution across varying terrains. Precipitation also played a significant, albeit complex, role. Interestingly, the relationship between precipitation and abundance indicated negative effects of rainfall, while lagged precipitation showed positive associations. This complexity likely reflects seasonal rainfall patterns and their influence on larval habitat availability. Similarly, Morin and Comrie [68] used the Dynamic Mosquito Simulation Model (DyMSiM) to show that the interaction between temperature and precipitation drives mosquito dynamics differently in California and Florida. Valdez et al. [69] further revealed that not only total rainfall, but also the number of rainy days and daily variability, strongly influence mosquito abundance. Our findings corroborate these earlier studies and extend them by explicitly testing whether accounting for spatial weather heterogeneity improves the performance of a mechanistic model.

Although the cluster-level model explains key features of the mosquito population dynamics at smaller spatial scales, aggregating cluster-level predictions to the county scale did not improve inference. A possible contributing reason for this contradiction is that the data we used per mosquito trap (PRISM) interpolates weather patterns across space on the basis of sparse station networks and provides 4 km × 4 km grid raster data. In Arizona, localized monsoon events produce intense, short-duration rainfall that varies significantly over short distances. PRISM’s data resolution may smooth these extremes, missing key signals needed for accurate prediction [70, 71]. Future studies could consider deploying ground-based weather stations near the mosquito traps to obtain high-resolution climate data. This could enhance the accuracy of fine-scale model predictions. Moreover, model error may accumulate during the aggregation process, particularly when fit to clusters with small sample sizes. Although the cluster-aggregated model did not improve county-wide predictions, it remains valuable for local inference and public health planning. Local-level fits can inform targeted mosquito control strategies and enhance situational awareness.

In our analysis, we aggregated cluster-level predictions to the county scale using a simple unweighted sum of predicted abundances across clusters. This approach directly parallels how observed mosquito abundance data are aggregated (summing trap counts across all traps) and allows for a fair comparison between the cluster-level and county-level modeling approaches. However, alternative aggregation schemes could potentially improve predictive performance for county-level forecasting applications. For instance, cluster-specific predictions could be weighted by factors such as: (1) ZCTA area or the number of traps within each cluster, to account for spatial sampling effort; (2) average mosquito abundance in each cluster, to emphasize regions with higher baseline populations; (3) model fit quality for each cluster, to give more weight to clusters where the model performs well; or (4) sociodemographic and infrastructure factors (human population density, wastewater system design), to prioritize areas with higher public health relevance and account for urban infrastructure affecting mosquito habitat [72–74]. Exploring such weighted aggregation schemes could be particularly valuable for operational forecasting systems, where the goal is to predict county-level disease risk rather than simply test whether spatial weather heterogeneity matters for mosquito dynamics. Additionally, hierarchical Bayesian approaches could simultaneously estimate both cluster-specific and county-level parameters while explicitly modeling the relationships between them, potentially improving both local and aggregate predictions. Future work should explore whether weighted aggregation or hierarchical modeling frameworks improve predictive accuracy for mosquito abundance forecasting at multiple spatial scales.

In our framework, we estimated a shared climate-response function for the mosquito population growth rate across all the clusters—the quadratic effect of temperature and the exponentially saturating effect of accumulated precipitation. Given that this same functional response explains the data across clusters, this provided evidence that mosquito subpopulation growth rates responded similarly to weather despite differing average abundances. However, the current approach did not account for mechanisms that may explain the average differences in abundance across clusters. Future research should explore how landscape features, water availability, or urban infrastructure might explain baseline variation in mosquito abundances. Avian factors such as bird abundance, nesting, and communal roosting behavior could play a key role, as these influence vector–host contact rates and amplification of pathogens such as West Nile virus in urban settings, with communal roosts often serving as focal points for heightened mosquito–host interactions [75–77]. Similarly, vegetative landcover (e.g., tree canopy or irrigated vegetation) is known to shape mosquito habitat suitability and abundance in desert urban environments such as those in Arizona, where greater tree cover or green spaces can support adult resting sites and larval development, whereas bare or impervious surfaces may limit them [78, 79].

We also encountered six ZIP codes with unusually high mosquito counts, especially in spring, which the model failed to capture even when fit separately. These outlier patterns were not explained by temperature or 30-day precipitation trends, indicating that local landscape effects such as standing water accumulation may be at play. Investigating these features—e.g., water-retaining infrastructure, wetlands, or irrigation systems—could yield important insights. Future work could incorporate additional covariates to explain baseline mosquito growth rates, such as proxies for standing water, land use, and hydrological features. High-resolution, daily updated data on these variables could better capture early season surges in abundance. Additionally, spatial movement of mosquitoes—whether natural or human-facilitated—should be considered in spatially explicit models.

Spatial processes are known to structure ecological and epidemiological dynamics, influencing everything from vector distribution to disease transmission and species interactions [65, 80]. Invasive species spread, habitat fragmentation, and local resource competition all highlight the importance of spatial structure in population persistence and disease risk [81]. By explicitly incorporating spatial heterogeneity, our model represented a step forward in mosquito modeling efforts. As weather forecasting capabilities continue to improve, spatially structured models will be critical for translating environmental changes into actionable public health responses.

Our model effectively captured seasonal abundance patterns without explicitly modeling behavioral mechanisms such as estivation. The rapid increases in abundance following monsoon rains could reflect either: (1) rapid population growth due to increased larval habitat availability, as represented in our model, or (2) synchronized oviposition by quiescing females when oviposition sites become available, as observed in other *Culex* species [82–85]. Disentangling these mechanisms would require additional data on mosquito physiological state and behavior throughout the season, which was beyond the scope of this study.

Climate change is reshaping ecosystems and disease risk globally [16–18]. Rising temperatures and altered precipitation patterns create more favorable conditions for mosquito proliferation and disease transmission [86, 87]. Accurate models that incorporate both temporal and spatial heterogeneity are essential to predict these dynamics and guide interventions.

## Conclusions

Our study demonstrated the value of spatially resolved climate-driven models for understanding mosquito population dynamics. Although challenges remain in scaling predictions and capturing outlier behavior, this work laid a foundation for more refined, localized modeling efforts that can enhance mosquito control and public health preparedness in a changing climate.

## Supplementary Information

Additional file 1. **Text S1**. Some details about outlier ZCTAs. **Text S2**. Temperature clustering analysis and justification for using precipitation only. **Text S3**. Hierarchical model inference across clusters. **Text S4**. Formulation of likelihood function using negative binomial probability distribution. **Text S5**. Interpretation of the outliers' ZCTAs results. **Text S6**. Model evaluation for 2 clusters. **Fig. S1**. Temperature and precipitation response functions used in the model. We used 2014 temperature and precipitation data from PRISM. **Fig. S2**. Comparison of clustering prediction, real data, and county prediction for 2 clusters. **Fig. S3**. A spatio-temporal plot of mosquito abundance per ZCTA. **Fig. S4**. Four cluster result and relationship between estimated baseline mosquito population growth rate and average mosquito abundance across clusters. **Fig. S5**. Functional PCA variance explained by the first four principal components. **Fig. S6**. Daily temperature patterns for five clusters in 2014–2016. Each panel shows the mean temperature trajectory across the year for clusters identified from the corresponding year’s data. **Fig. S7**. Spatial standard deviation of temperature and precipitation across ZCTAs over time. **Fig. S8**. Quantitative comparison of spatial heterogeneity between temperature and precipitation. **Fig. S9**. 9 Cluster prediction. **Fig. S10**. 9 Cluster 2013 prediction. **Fig. S11**. 9 Cluster 2017 prediction. **Fig. S12**. 2 Cluster prediction. **Figs. S13-S17**. Histograms of marginal posterior draws of parameters. **Figs. S18-S22**. Pairs plot displaying joint posterior draws of parameters. **Fig. S23**. Predicted versus actual for all clusters. **Table S1**. Parameter estimated value and its prior for one cluster. **Table S2**. Parameter estimated value and its prior for more than one cluster. **Table S3**. Model evaluation at cluster level for five clusters. **Table S4**. Model evaluation at cluster level for nine clusters.

## Data Availability

Data supporting the main conclusions of this study are included in the manuscript.
