# Supplementary material for: Accounting for spatial variation in weather factors predicts spatial variations in Culex quinquefasciatus abundance in the Desert Southwest
Source: Parasit Vectors. 2026 Mar 11;19:170. doi: 10.1186/s13071-026-07326-z (PMC13088551; doi:10.1186/s13071-026-07326-z)
Supplement: Supplementary file 1 — Additional file 1. Text S1. Some details about outlier ZCTAs. Text S2. Temperature clustering analysis and justification for using precipitation only. Text S3. Hierarchical model inference across clusters. Text S4. Formulation of likelihood function using negative binomial probability distribution. Text S5. Interpretation of the outliers' ZCTAs results. Text S6. Model evaluation for 2 clusters. Fig. S1. Temperature and precipitation response functions used in the model. We used 2014 temperature and precipitation data from PRISM. Fig. S2. Comparison of clustering prediction, real data, and county prediction for 2 clusters. Fig. S3. A spatio-temporal plot of mosquito abundance per ZCTA. Fig. S4. Four cluster result and relationship between estimated baseline mosquito population growth rate and average mosquito abundance across clusters. Fig. S5. Functional PCA variance explained by the first four principal components. Fig. S6. Daily temperature patterns for five clusters in 2014–2016. Each panel shows the mean temperature trajectory across the year for clusters identified from the corresponding year’s data. Fig. S7. Spatial standard deviation of temperature and precipitation across ZCTAs over time. Fig. S8. Quantitative comparison of spatial heterogeneity between temperature and precipitation. Fig. S9. 9 Cluster prediction. Fig. S10. 9 Cluster 2013 prediction. Fig. S11. 9 Cluster 2017 prediction. Fig. S12. 2 Cluster prediction. Figs. S13-S17. Histograms of marginal posterior draws of parameters. Figs. S18-S22. Pairs plot displaying joint posterior draws of parameters. Fig. S23. Predicted versus actual for all clusters. Table S1. Parameter estimated value and its prior for one cluster. Table S2. Parameter estimated value and its prior for more than one cluster. Table S3. Model evaluation at cluster level for five clusters. Table S4. Model evaluation at cluster level for nine clusters. [file 13071_2026_7326_MOESM1_ESM.pdf]

Supplement for “Accounting for spatial variation in climatic factors predicts spatial variations in mosquito abundance in the desert southwest”

Oshinubi et al.

February 5, 2026

In this supplementary material, we present some additional information, plots, and tables from our analysis. In Section 1, we present some details about outlier ZIP Code Tabulation Areas (ZCTAs). In Section 2, we present Temperature clustering analysis and justification for using precipitation only. In Section 3, we present hierarchical model inference across clusters we developed, which helped us to estimate unique parameters for each cluster. In Section 4, we present the formulation of our likelihood function using negative binomial probability distribution. In Section 5, we provided the interpretation of our results of the outliers ZCTAs. In Section 6, we present the model evaluation for 2 clusters. In Section 7, we present supplementary tables. In Section 8, we present some additional figures generated from our analysis.

1 Outlier ZCTA

During initial data exploration, we noticed that a small number of the 115 ZCTAs contained much higher average mosquito abundances. To quantitatively define ’outlier’ ZCTA in our data set, we divided the training data set into two temporal windows, the first half and second half of each year, which tend to show unique patterns in mosquito abundances. We then calculated the 95% quantiles of mosquito abundance across the 115 ZCTAs for the first half and second half of the year across the three-year data set. We considered ZCTAs as ’outliers’ if their average abundance was higher than the 95% abundance quantile in either the first or second half of the year. From this, we identified 6 outlier ZCTAs, 5 of which had high average abundance in both time periods, whereas one outlier had high average abundance in the second half of the year (see Figure S3 in the supplementary file). In fact, across the three years of training data, these 6 ZCTAs account for a total of 42.5% of the observed mosquito abundance.

Importantly, these six outliers also contribute to unique patterns in the mosquito abundance time series not observed across the other 109 ZCTAs (Figure 1b in the main text). The outliers show large spikes in abundance in the spring, which do not seem to correlate with accumulated precipitation. We discuss hypotheses for this phenomenon in more detail later, but briefly, these abundance increases in outlier locations early in the year are likely due to standing water that has accumulated over the winter months, rather than real-time precipitation patterns. Therefore, in our analysis, we evaluated how the inclusion or exclusion of these outliers impacted our model fitting and out-of-sample prediction.

2 Temperature clustering analysis and justification for using precipitation only

We initially explored clustering ZCTAs using both temperature and precipitation data to identify areas with similar climate conditions that might drive spatial variation in mosquito abundance. However, preliminary analyses revealed that temperature showed minimal spatial variation across Maricopa County compared to precipitation, making it unsuitable as a primary clustering variable. Here we provide detailed justification for our decision to cluster ZCTAs based on precipitation alone.

We applied functional data analysis (FDA) and multiple clustering algorithms to both temperature and precipitation time series data for all ZCTAs across Maricopa County during the training period (2014-2016). We used the fda package in R to perform functional principal component analysis (fPCA) on both temperature and precipitation time series. Time series were smoothed using Fourier basis functions with 1,096 basis functions to match the three-year daily time series. We examined the proportion of variance explained by the first four principal components for each climate variable to assess the degree of spatial heterogeneity. We tested multiple network-based clustering algorithms on the functional principal component scores, including Louvain clustering (with resolution parameter testing: 1.0, 1.001, 1.005, 1.01, 1.05, 1.1), Spinglass clustering, Walktrap clustering, and Infomap clustering. For each algorithm, we varied the Euclidean distance radius threshold from 1 to 14 km to define network edges between spatially proximate ZCTAs with similar climate patterns. We also applied the FunFEM package to directly cluster the smoothed functional data, testing 5 to 10 clusters for both temperature and precipitation independently, as well as for combined temperature and precipitation data.

To quantitatively assess spatial heterogeneity, we calculated the spatial standard deviation (SD) across ZCTAs for each day of the three-year study period (1,096 days). The spatial SD measures how much climate values vary across different locations on the same day, with higher values indicating greater spatial heterogeneity. We also calculated the coefficient of variation (CV = spatial SD / mean) to compare relative spatial heterogeneity between the two climate variables, which accounts for differences in units and magnitudes.

Functional PCA of temperature data revealed that the first principal component explained more than 98% of variance across all years: 98.26% in 2014, 98.32% in 2015, 98.27% in 2016, and 98.10% for the combined 2014-2016 period (see Figure S5 (right) in the supplementary file). This indicates that temperature patterns are nearly identical across all ZCTAs in Maricopa County, with spatial differences accounting for less than 2% of total variance (see Figure S6 in the supplementary file). The remaining principal components (PC2-PC4) each explained less than 1% of variance, further confirming the lack of meaningful spatial heterogeneity in temperature. In contrast, precipitation showed substantial spatial heterogeneity (see Figure S5 (left) in the supplementary file). The first four principal components combined explained only modest proportions of total precipitation variance: 97.6% in 2014 (45.6%, 34.7%, 15.0%, 2.1% for PC1-4 respectively), 87.9% in 2015 (41.3%, 29.1%, 12.2%, 5.7%), 94.4% in 2016 (70.2%, 14.4%, 6.7%, 3.1%), and 84.4% for the combined period (36.4%, 27.6%, 15.0%, 5.4%). The more distributed variance across multiple principal components indicates meaningful spatial variation in precipitation patterns, including both magnitude and some seasonal timing differences.

Temperature showed minimal spatial variation, with a mean spatial SD of 0.64°C (mean spatial range: 4.05°C across ZCTAs) over the 1,096-day study period (Supplementary Figure S7 (bottom)). This represents a coefficient of variation (CV) of 0.021, indicating that spatial differences account for only 2.1% of the mean temperature value (29.84°C). In contrast, precipitation showed substantially greater relative spatial heterogeneity, with a mean spatial SD of 0.11 mm (mean spatial range: 0.54 mm) and a CV of 0.30 (Supplementary Figure S7 (top)). The coefficient of variation for precipitation was 14-fold larger than for temperature (0.30 vs. 0.021), confirming that precipitation exhibits substantially greater relative spatial heterogeneity despite having a smaller absolute SD. This difference reflects the fact that precipitation is highly episodic (with many zero values) and spatially variable, while temperature varies smoothly across space with minimal heterogeneity relative to its mean (Supplementary Figure S8).

When we applied network-based clustering algorithms (Louvain, Spinglass, Walktrap, Infomap) to temperature PCA scores, the resulting clusters exhibited three major problems. First, cluster assignments were highly unstable, changing dramatically with minor parameter adjustments (e.g., changing the Louvain resolution parameter from 1.0 to 1.001 resulted in completely different cluster configurations). Second, the clusters were not geographically meaningful—they did not correspond to spatial proximity, elevation gradients, or known urban-rural climate differences in the county. Third, because more than 98% of variance is shared across all ZCTAs, any clustering was necessarily based on less than 2% residual variation, which likely represents measurement noise rather than true climate differences. This contrasts sharply with precipitation-based clustering, which produced stable, geographically coherent clusters that corresponded to known elevation and urban-rural gradients in the county (see Fig. 1b in main text). Combined temperature and precipitation clustering was redundant with precipitation-only clustering. When we in-

cluded both temperature and precipitation in multivariate clustering analyses, the results were nearly identical to precipitation-only clustering. ZCTA assignments agreed in more than 95% of cases, with minor differences occurring only in boundary ZCTAs between adjacent clusters. This redundancy occurred because temperature variation across ZCTAs is negligible (as shown in Fig. 2a of the main text), such that precipitation dominates any multivariate Euclidean distance metric. Adding temperature to the clustering algorithm simply introduced noise without improving cluster separation or providing additional information about spatial climate heterogeneity.

These results are consistent with visual inspection of Fig. 2 in the main manuscript, which shows that temperature time series from different ZCTAs are nearly superimposed (Fig. 2a), whereas precipitation time series show clear differences in magnitude and some differences in timing across clusters (Fig. 2b).

Based on these formal quantitative analyses, we concluded that temperature exhibits insufficient spatial variation across Maricopa County to serve as a useful clustering variable. Multiple independent analyses support this conclusion: (1) functional PCA showing $> 98\%$ of variance explained by PC1, (2) spatial standard deviation analysis showing a coefficient of variation 14-fold lower than precipitation, and (3) clustering algorithms producing unstable, non-meaningful results. Precipitation, in contrast, shows substantial spatial heterogeneity across all metrics and produces stable, geographically meaningful clusters when subjected to functional clustering algorithms. Including temperature in clustering analyses adds no information and only introduces computational complexity, as the results are redundant with precipitation-only clustering. Therefore, we used precipitation-only clustering for all analyses reported in the main manuscript. This decision was based on rigorous quantitative assessment of spatial variance structure through functional PCA, spatial standard deviation analysis, and multiple clustering algorithms, not merely visual inspection of time series plots.

3 Hierarchical model inference across clusters

For the three clustered data partitions, we estimated some parameters across the clusters, while some parameters were estimated uniquely to each cluster. Specifically, it seems most biologically reasonable to assume that the effects of temperature and precipitation (i.e., how mosquito population growth rate responds to fluctuations in climate) do not vary spatially. However, the average abundance of mosquitoes does vary across space. Therefore, we allowed the baseline population growth rate, ν, to vary across clusters (i.e., estimating ν_k across k clusters), while holding all other model parameters constant across the clusters.

To do this efficiently, though, we first had to explore how baseline growth rate ν_k was related to average mosquito population size, because mathematically, ν_k should scale approximately linearly with average abundance. Therefore, we performed some preliminary MCMC explorations to develop an appropriate hierarchical prior distribution for ν_k. We used k-means clustering to divide the ZCTA into 4 clusters, approximately dividing the county into quadrants, each with quite different average mosquito abundances (see Figure S4a in the supplementary file). Then, we ran four independent MCMC analyses, one on each cluster, to estimate all the model parameters, including ν, using very broad and vague priors. Finally, we conducted a regression of the posterior estimates of ν in each cluster (i.e., 4 values of ν) against the average mosquito abundance in each respective cluster (see Figure S4b in the supplementary file).

In the hierarchical MCMC, we then used the estimated regression parameters (intercept, slope, residual variance, and their covariance matrix) to construct a multivariate normal prior. Specifically, at each MCMC iteration, we randomly drew from a multivariate normal to generate proposal parameters of the linear model that explains the relationship between ν and average mosquito abundance. We then use these proposed linear model parameters to generate a proposal vector of ν_k, based on the clusters' average mosquito abundances. This way, we could use the same multivariate normal prior regardless of how many clusters we used in the data partition. Additionally, this hierarchical inference method was much more efficient than estimating each ν_k as independent parameters in the Metropolis-Hastings MCMC. All prior distributions for the model parameters are provided in Tables S1–S2 of the supplementary material. To evaluate the parameter estimation results, we include histograms (Supplementary Figures S13–S17) illustrating the posterior distributions of each parameter. Additionally, we present pairs plots (Supplementary Figures S18–S22) to explore relationships and potential correlations between parameters. Finally,

a 1:1 plot (Supplementary Figure S23) is used to visually assess the agreement between observed and predicted values, providing insight into the predictive performance of the model.

To assess whether accounting for spatial heterogeneity in weather improved county-level predictions, we aggregated cluster-level model predictions to the county scale and compared these aggregated predictions to the county-level model fit. Importantly, we did not generate a single 'county-level' parameter set from the cluster-specific parameters. Instead, for each draw from the joint posterior distribution, we generated predictions for each cluster using its cluster-specific parameters, then summed the predicted abundances across all clusters for each week to obtain county-wide predictions. This approach preserves the cluster-specific parameter estimates and weather effects while allowing us to evaluate whether modeling spatial heterogeneity improves aggregate predictions. We used a simple unweighted sum (rather than weighted aggregation by factors such as ZCTA area, trap density, or model fit quality) to provide a direct comparison with the county-level model, which similarly sums observed abundances across all traps. This unweighted approach allows us to isolate the effect of accounting for spatial weather heterogeneity on prediction accuracy without introducing additional assumptions about how to weight different spatial units.

4 Likelihood Formulation

The likelihood function is defined using a negative binomial probability distribution to accommodate over-dispersion in observed mosquito abundance data across multiple spatial clusters. The likelihood function is constructed to evaluate how well the simulated mosquito abundance matches observed values across clusters. The abundance dynamics are modeled by a temperature- and precipitation-driven ordinary differential equation (ODE), and the likelihood aggregates over all clusters.

Let $X_i^{(k)}$ denote the observed mosquito abundance at time point i in cluster k, and let $\gamma_i^{(k)}$ denote the corresponding model-predicted abundance obtained by numerically integrating the ODE climate-forced mathematical model in Section 2.2 in the main text for each cluster k. To allow for overdispersion, the observation model assumes that each $X_i^{(k)}$ follows a negative binomial distribution:

$$X_i^{(k)} \sim \mathrm{NegBin}(\gamma_i^{(k)}, r), \tag{1}$$

where $r = 1/inv\_disp$ is the size (shape) parameter and inv_disp is the estimated inverse-dispersion parameter. The negative binomial probability mass function (PMF) for this parameterization is:

$$\Pr(X = j) = \frac{\Gamma(j+r)}{j!\,\Gamma(r)} \left(\frac{r}{r+\gamma}\right)^r \left(\frac{\gamma}{r+\gamma}\right)^j, \tag{2}$$

for $j \in \mathbb{N}$, $\gamma > 0$, and $r > 0$.

The log-likelihood contribution from the cluster k is

$$\mathscr{L}^{(k)} = \sum_{i \in \mathscr{T}_k} \log \left[\mathrm{NegBin}(X_i^{(k)} \mid \gamma_i^{(k)}, 1/inv\_disp) \right], \tag{3}$$

where $\mathscr{T}_k$ is the set of observation times for cluster k.

The total log-likelihood across all clusters is

$$\mathscr{L}_{\mathrm{total}} = \sum_{k=1}^{n_{\mathrm{cluster}}} \mathscr{L}^{(k)}. \tag{4}$$

In implementation, a small positive value (e.g., 10^{-5}) is substituted for any negative or zero $\gamma_i^{(k)}$ values to ensure numerical stability when evaluating the log-likelihood.

The likelihood is evaluated using parameters grouped as follows:

- **Fixed parameters** $(\alpha, \phi, T_{\min}, T_{\max}, inv\_disp)$,

- **Cluster-specific parameters** $v_1, \ldots, v_{(n_{\text{cluster}})}$.

The cluster-specific v_k values are drawn from a linear regression model with mean mosquito abundance as a predictor:

$$v_k = \beta_0 + \beta_1 \cdot A_k + \varepsilon_k, \quad \varepsilon_k \sim \mathcal{N}(0, \sigma^2), \tag{5}$$

where A_k is the mean observed abundance for cluster k. The regression parameters $(\beta_0, \beta_1, \sigma)$ are estimated via maximum likelihood and used to generate v_k values in the MCMC proposal step.

This formulation allows the model to capture heterogeneous dynamics across spatial clusters while accommodating observational over-dispersion through the negative binomial framework.

5 Evaluating outlier zip codes

When we include the six outlier ZCTAs in the training data, our qualitative comparisons between county- and cluster-level models are unchanged, such that the county-level modeling is more parsimonious and the dynamical model fits well to the data in most parts of the year. However, these outlier ZCTAs reveal important areas for model improvement (supplementary Figure S2 and Figure S12 (first row)). For the outlier ZCTAs, the RMSE for the fitted data is 308.7358, and the WAIC value is 2017.8, while the predicted RMSE values for 2013 and 2017 are 298.3209 and 189.0854, respectively. When aggregating two clusters—comprising the county level without the 'outlier' ZCTAs and the 'outlier' ZCTAs—the RMSE for the fitted data is 434.7729. The corresponding RMSE values for the predicted data in 2013 and 2017 are 459.0103 and 484.355, respectively. What becomes obvious is that the model fails to predict large early-year increases in mosquito abundance in these six ZCTAs. These early-year abundances do not seem to be explained by the climate data that we provide the model. This begs the question as to why mosquito abundances reach very large numbers in the early season for these specific ZCTAs. This consistent underperformance during the early year suggests a potential "spring effect" in these locations. A closer examination of these ZCTAs reveals that they are located near agricultural lands, irrigation fields, and river runoff areas, suggesting that unique environmental factors in these regions may influence the mechanisms by which precipitation and/or temperature affect mosquito abundance during the spring season.

6 Evaluating 2 cluster (outliers ZCTAs and other ZCTAs)

In this section, we examine the clustering results when we identified two distinct clusters during our initial data exploration, namely, cluster 1 (outlier ZCTAs) and cluster 2 (other ZCTAs). For the 2-cluster subdivision, the predicted RMSE values for Cluster 1 and Cluster 2 are 313.807 and 254.352, respectively, while the WAIC for Cluster 1 and Cluster 2 are 2124.5 and 3722.6, respectively. For the fitted data in 2013, the RMSE values are 291.598 for Cluster 1 and 312.559 for Cluster 2. In 2017, the RMSE values for Cluster 1 and Cluster 2 were 169.330 and 505.083, respectively. In supplementary Figure S12, we present 2 cluster predictions.

7 Supplementary Tables

Table 1: Parameter estimated value and its prior for one cluster

Parameters	Prior				
$\begin{pmatrix}\beta_0\\ \beta_1\\ \sigma\end{pmatrix}$	$\mathcal{N}(\begin{pmatrix}\beta_0\\ \beta_1\\ \sigma\end{pmatrix}, \begin{pmatrix}0.05937776\\ 0.002153349\\ 0.09031871\end{pmatrix}, \begin{pmatrix}1.262493e^{-04} & -4.534549e^{-07} & -2.767479e^{-09}\\ -4.534549e^{-07} & 1.942469e{-}09 & 1.035168e^{-11}\\ -2.767479e^{-09} & 1.035168e^{-11} & 1.018966e^{-05}\end{pmatrix})$				
$(\nu_k)_{k=1...9}$	Normal$(\nu_k, \beta_0+\beta_1 * A_k, \sigma * \tau)$, τ is 1 for k > 2 and 20 for k = 1,2				
County without 'outliers' ZCTAs					
	Median	25.0%	97.5%	Prior	Rhat
$\begin{pmatrix}\beta_0\\ \beta_1\\ \sigma\end{pmatrix}$	$\begin{pmatrix}0.02976152\\ 0.002246532\\ 0.07624972\end{pmatrix}$	$\begin{pmatrix}0.02433612\\ 0.002226002\\ 0.07464232\end{pmatrix}$	$\begin{pmatrix}0.04552954\\ 0.002314214\\ 0.10095124\end{pmatrix}$	-	$\begin{pmatrix}1.000188\\ 1.004348\\ 1.005444\end{pmatrix}$
ν_1	1.719676	1.560024	2.402226	-	1.01624
$Tmin$	18.95258	18.56927	19.89583	Uniform($Tmin$, 15,23)	1.010856
$Tmax$	45.21634	44.42262	49.69285	Uniform($Tmax$, 40,55)	1.013312
α	1.4823936	1.2838298	2.1524761	Uniform(α, 0.25,2.5)	1.019886
ϕ	1.3716845	1.1376146	2.7305792	Uniform(ϕ, 0.9,10)	1.014801
inv_disp	0.3554648	0.3283849	0.4851967	Uniform(inv_disp, 0.1,0.8)	1.003655
'outliers' ZCTAs					
$\begin{pmatrix}\beta_0\\ \beta_1\\ \sigma\end{pmatrix}$	$\begin{pmatrix}0.03976153\\ 0.002246533\\ 0.08624973\end{pmatrix}$	$\begin{pmatrix}0.03433613\\ 0.002226003\\ 0.08464233\end{pmatrix}$	$\begin{pmatrix}0.05552955\\ 0.002314215\\ 0.10095125\end{pmatrix}$	-	$\begin{pmatrix}1.000336\\ 1.005444\\ 1.004348\end{pmatrix}$
ν_1	1.675081	1.539098	2.214737	-	1.01692
$Tmin$	21.19682	20.86893	21.86433	Uniform($Tmin$, 15,23)	1.019605
$Tmax$	42.85949	42.38078	46.80677	Uniform($Tmax$, 40,55)	1.028547
α	0.9169440	0.6672233	1.9362353	Uniform(α, 0.25,2.5)	1.040783
ϕ	1.0858819	0.9859543	2.3321332	Uniform(ϕ, 0.9,10)	1.014189
inv_disp	0.7014340	0.6563753	0.7951554	Uniform(inv_disp, 0.1,0.8)	1.004445

Table 2: Parameter estimated value and its prior for more than one cluster

Parameters	2 clusters				
	Median	25.0%	97.5%	Prior	Rhat
$\begin{pmatrix}\beta_0\\ \beta_1\\ \sigma\end{pmatrix}$	$\begin{pmatrix}0.04976155\\ 0.002246531\\ 0.09624977\end{pmatrix}$	$\begin{pmatrix}0.04433612\\ 0.002226002\\ 0.09464232\end{pmatrix}$	$\begin{pmatrix}0.06552957\\ 0.002314210\\ 0.10095125\end{pmatrix}$	-	$\begin{pmatrix}1.003371\\ 1.005444\\ 1.004348\end{pmatrix}$
v_1	1.1475184	0.9767011	1.9081644	-	1.320659
v_2	1.6285085	1.4167489	2.7619224	-	1.297385
$Tmin$	19.78218	19.50840	20.51363	Uniform($Tmin$, 15,23)	1.088686
$Tmax$	44.47620	43.91322	47.81152	Uniform($Tmax$, 40,55)	1.093162
α	0.9694519	0.7066512	1.8108229	Uniform(α, 0.25,2.5)	1.190052
ϕ	1.0910817	0.9882769	1.8744850	Uniform(ϕ, 0.9,6)	1.05833
inv_disp	0.5408785	0.5129148	0.6304361	Uniform(inv_disp, 0.1,0.8)	1.001621
5 clusters					
$\begin{pmatrix}\beta_0\\ \beta_1\\ \sigma\end{pmatrix}$	$\begin{pmatrix}0.06341531\\ 0.002133807\\ 0.08550928\end{pmatrix}$	$\begin{pmatrix}0.05826092\\ 0.002114197\\ 0.08413671\end{pmatrix}$	$\begin{pmatrix}0.07795327\\ 0.002191405\\ 0.08968036\end{pmatrix}$	-	$\begin{pmatrix}1.000575\\ 1.000489\\ 1.000326\end{pmatrix}$
v_1	0.3014930	0.2818043	0.3627244	-	1.032145
v_2	0.4382690	0.4125237	0.5201658	-	1.026701
v_3	0.2591353	0.2430725	0.3131752	-	1.035533
v_4	0.1311577	0.1214776	0.1587472	-	1.033824
v_5	0.1712375	0.1593046	0.2086824	-	1.037363
$Tmin$	16.39452	16.21301	17.36529	Uniform($Tmin$, 15,23)	1.109313
$Tmax$	49.57801	48.67387	52.79773	Uniform($Tmax$, 40,55)	1.136697
α	1.865516	1.748551	2.145747	Uniform(α, 0.25, 2.5)	1.222095
ϕ	1.2131562	1.1118972	1.5750627	Uniform(ϕ, 0.9,6)	1.047742
inv_disp	0.4930550	0.4751286	0.5470297	Uniform(inv_disp, 0.1,0.8)	1.002397
9 clusters					
$\begin{pmatrix}\beta_0\\ \beta_1\\ \sigma\end{pmatrix}$	$\begin{pmatrix}0.05890547\\ 0.002162998\\ 0.08881471\end{pmatrix}$	$\begin{pmatrix}0.05377620\\ 0.002142091\\ 0.08730433\end{pmatrix}$	$\begin{pmatrix}0.07409803\\ 0.002223244\\ 0.09321634\end{pmatrix}$	-	$\begin{pmatrix}1.000442\\ 1.000309\\ 1.000327\end{pmatrix}$
v_1	0.2294917	0.2140709	0.2751476	-	1.071692
v_2	0.05781203	0.05320818	0.07176764	-	1.068338
v_3	0.06271807	0.05767068	0.07784730	-	1.077563
v_4	0.09522864	0.08782413	0.11563258	-	1.078966
v_5	0.1962258	0.1825582	0.2371645	-	1.089494
v_6	0.3926066	0.3672039	0.4624060	-	1.07284
v_7	0.07094355	0.06506929	0.08601582	-	1.096995
v_8	0.1296059	0.1199495	0.1573306	-	1.077736
v_9	0.09760678	0.09056399	0.11853778	-	1.079821
$Tmin$	12.13756	11.86880	13.32317	Uniform($Tmin$, 10.0, 17.0)	1.019512
$Tmax$	54.49941	54.10153	54.97587	Uniform($Tmax$, 40.0, 55.0)	1.023635
α	2.432134	2.353598	2.496935	Uniform(α, 0.25, 2.5)	1.09014
ϕ	0.8115820	0.7652920	0.9560820	Uniform(ϕ, 0.5, 2.0)	1.016278
inv_disp	0.6950292	0.6776585	0.7510000	Uniform(inv_disp, 0.1, 0.8)	1.003514

Table 3: Model evaluation at cluster level for five cluster

Model	Cluster1 (RMSE)	Cluster2 (RMSE)	Cluster3 (RMSE)	Cluster4 (RMSE)	Cluster5 (RMSE)
5 Cluster (2013)	53.980	116.430	61.840	259.500	66.280
5 Cluster (2014-2016)	80.240	104.150	64.860	35.210	40.620
5 Cluster (2017)	120.970	191.655	142.421	68.479	83.322

Table 4: Model evaluation at cluster level for nine cluster

Model / RMSE	Cluster1	Cluster2	Cluster3	Cluster4	Cluster5	Cluster6	Cluster7	Cluster8	Cluster9
9 Cluster (2013)	49.301	17.698	17.199	35.118	46.547	292.185	18.659	48.447	52.276
9 Cluster (2014-2016)	64.342	16.063	22.407	23.297	54.770	96.401	30.620	43.495	29.474
9 Cluster (2017)	69.260	21.112	18.949	62.809	135.524	161.591	60.480	42.767	84.735

8 Supplementary Figures

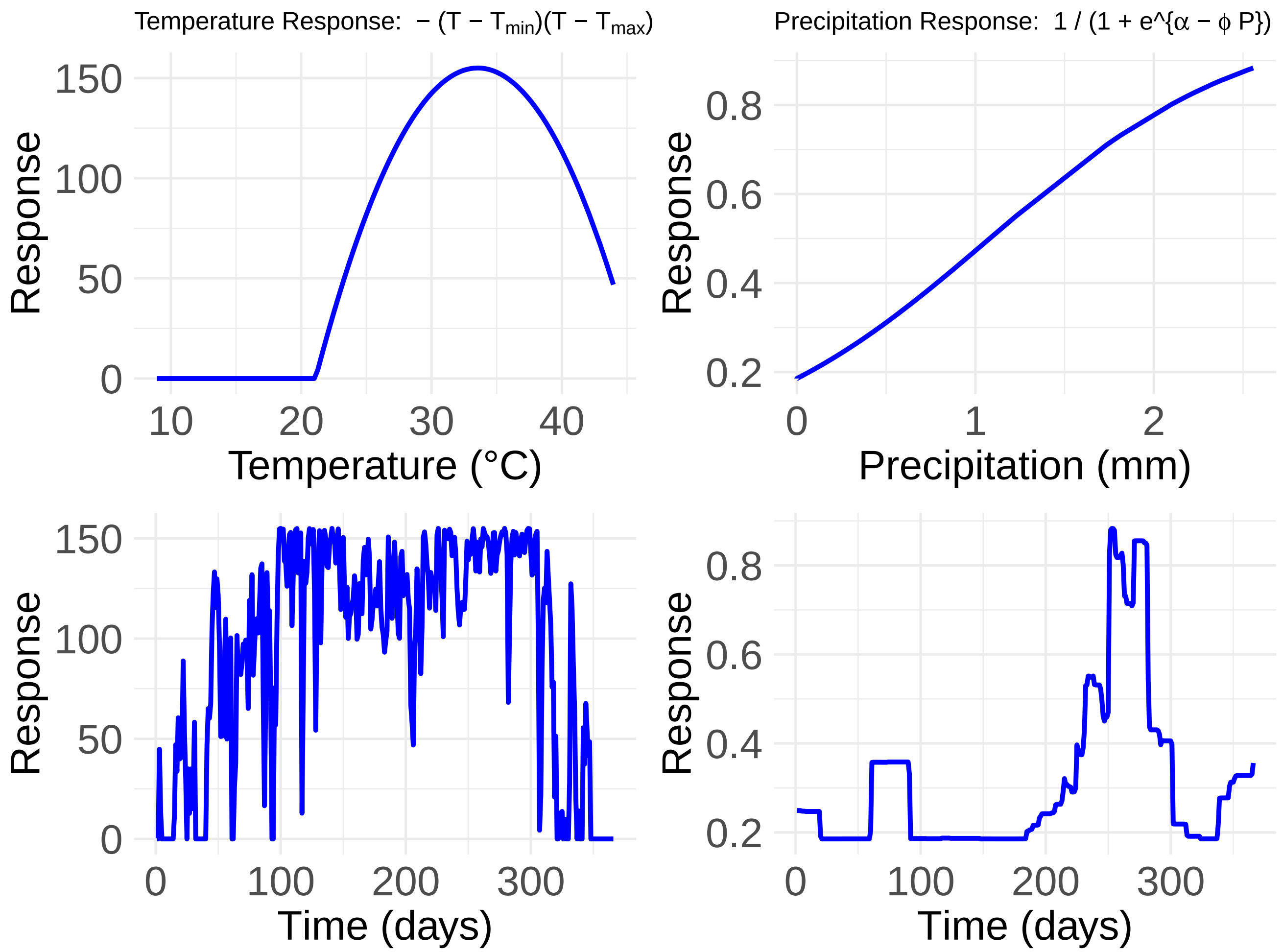

Figure 1: Temperature and precipitation response functions used in the model. We used 2014 temperature and precipitation data from PRISM. (**Top left**) Temperature response function defined as $-(T - T_{\text{min}})(T - T_{\text{max}})$, illustrating the parabolic relationship between temperature and suitability, with zero response below the minimum threshold T_{min} and above the maximum threshold T_{max}, and maximum response at intermediate temperatures. (**Top right**) Precipitation response function defined as $\frac{1}{1+\exp(\alpha - \phi P)}$, showing the sigmoidal increase in suitability with increasing daily precipitation, saturating at higher rainfall. (**Bottom left**) Time series of the temperature response across one year, showing variability in response driven by daily fluctuations in observed temperature. (**Bottom right**) Time series of the precipitation response across the same period, reflecting the nonlinear effects of rainfall pulses and dry periods on response values.

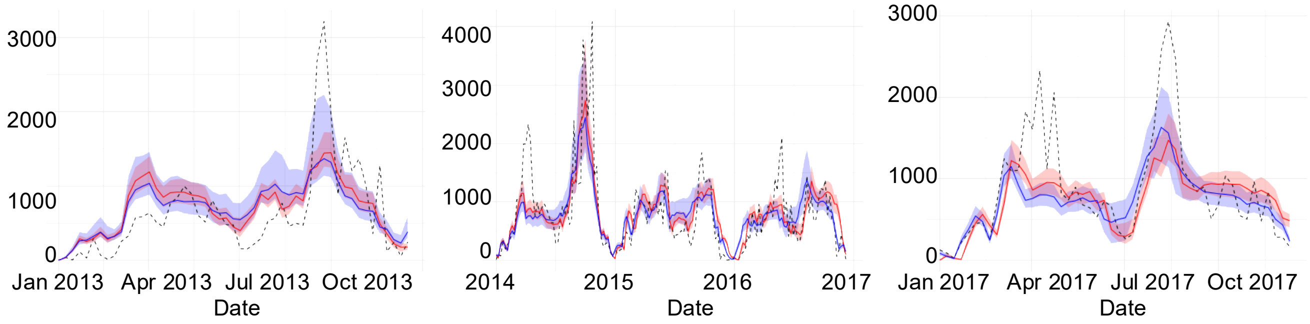

Figure 2: Comparison of clustering prediction, real data, and county prediction for 2 clusters. Shaded ribbons in blue indicate 95% credible intervals for county prediction, while shaded ribbons in red indicate 95% credible intervals for cluster sum prediction. The black dotted lines represent observed mosquito abundance data over time.

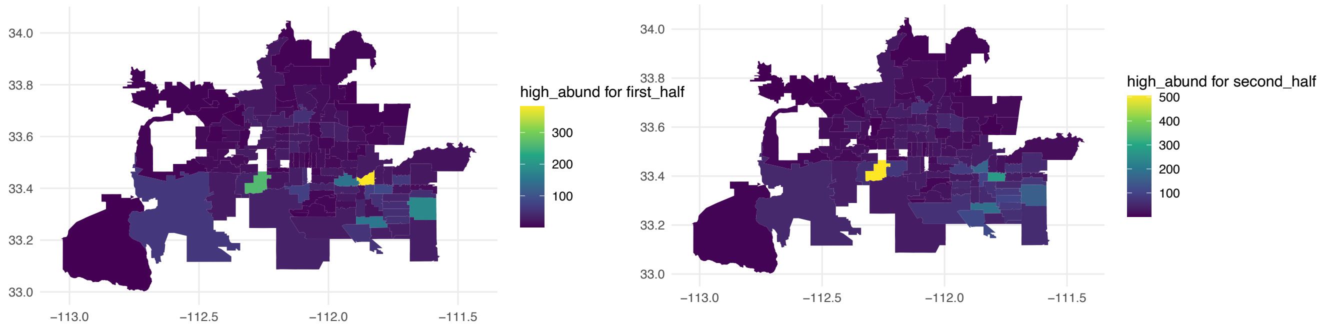

Figure 3: Left: A spatio-temporal plot of mosquito abundance per ZCTA for the first half of the year, with high abundance defined as the 97.5th percentile of total mosquitoes sampled. Right: A spatio-temporal plot of mosquito abundance per ZCTA for the second half of the year, with high abundance defined as the 97.5th percentile of total mosquitoes sampled.

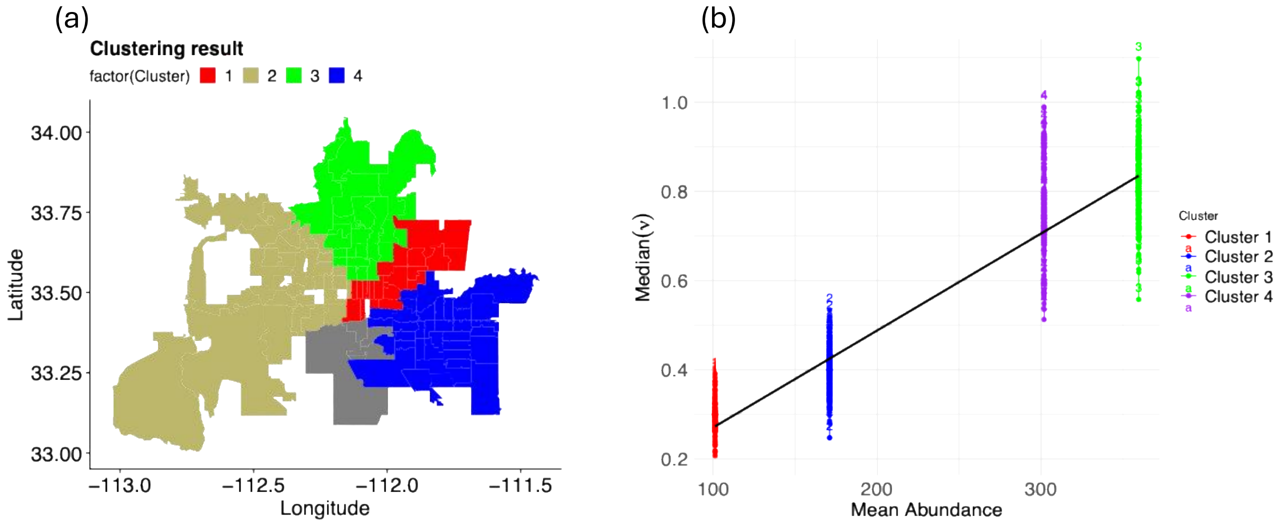

Figure 4: (a) 4 cluster result (b) Relationship between estimated baseline mosquito population growth rate (ν_k) and average mosquito abundance across clusters. Each point represents the posterior mean estimate of ν for one of the four spatial clusters obtained from independent MCMC analyses using vague priors. A linear regression line is shown, summarizing the trend used to define a hierarchical prior for ν_k in the main hierarchical MCMC analysis. This relationship supports the assumption that ν_k scales approximately linearly with average mosquito abundance across clusters.

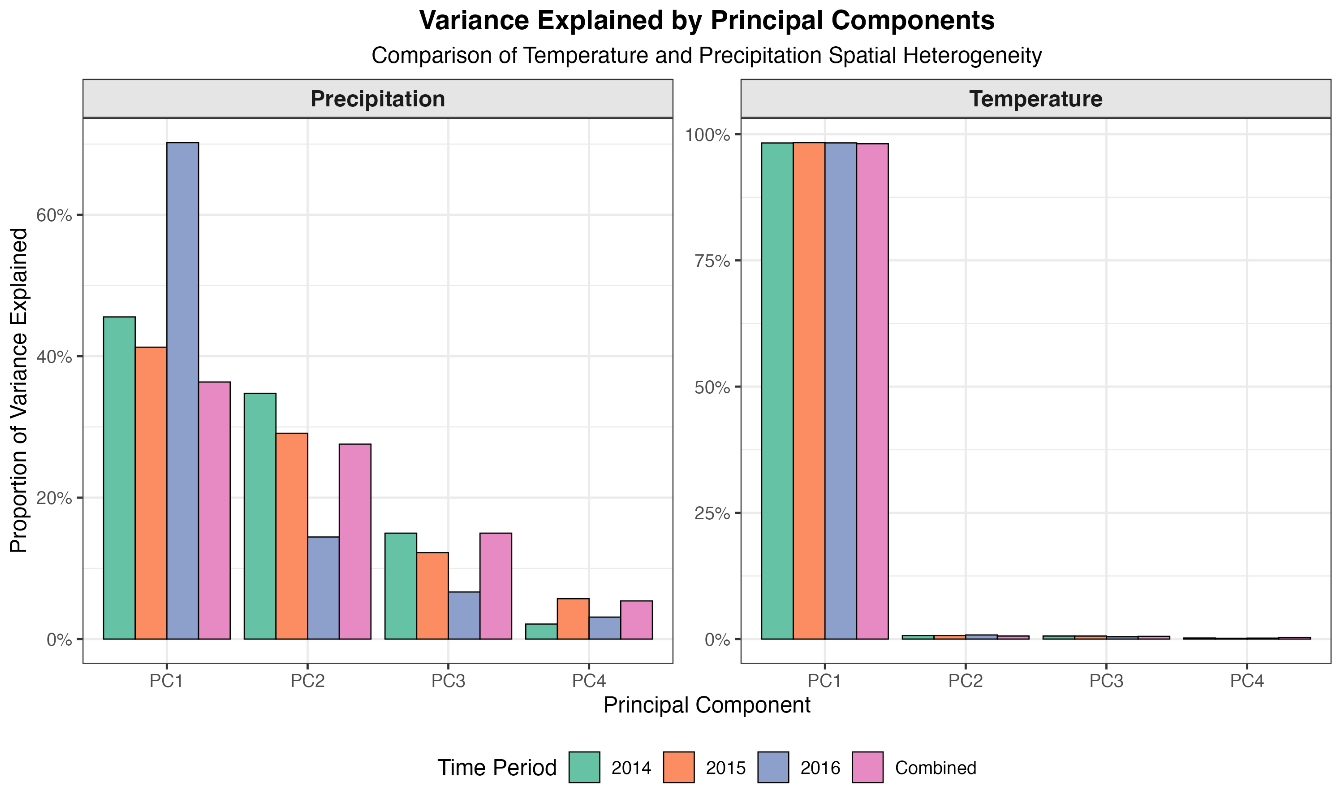

Figure 5: Functional PCA variance explained by the first four principal components for (Right) temperature and (Left) precipitation data across 2014-2016. Temperature PC1 explains $> 98\%$ of variance, indicating minimal spatial variation, while precipitation shows more distributed variance across multiple PCs.

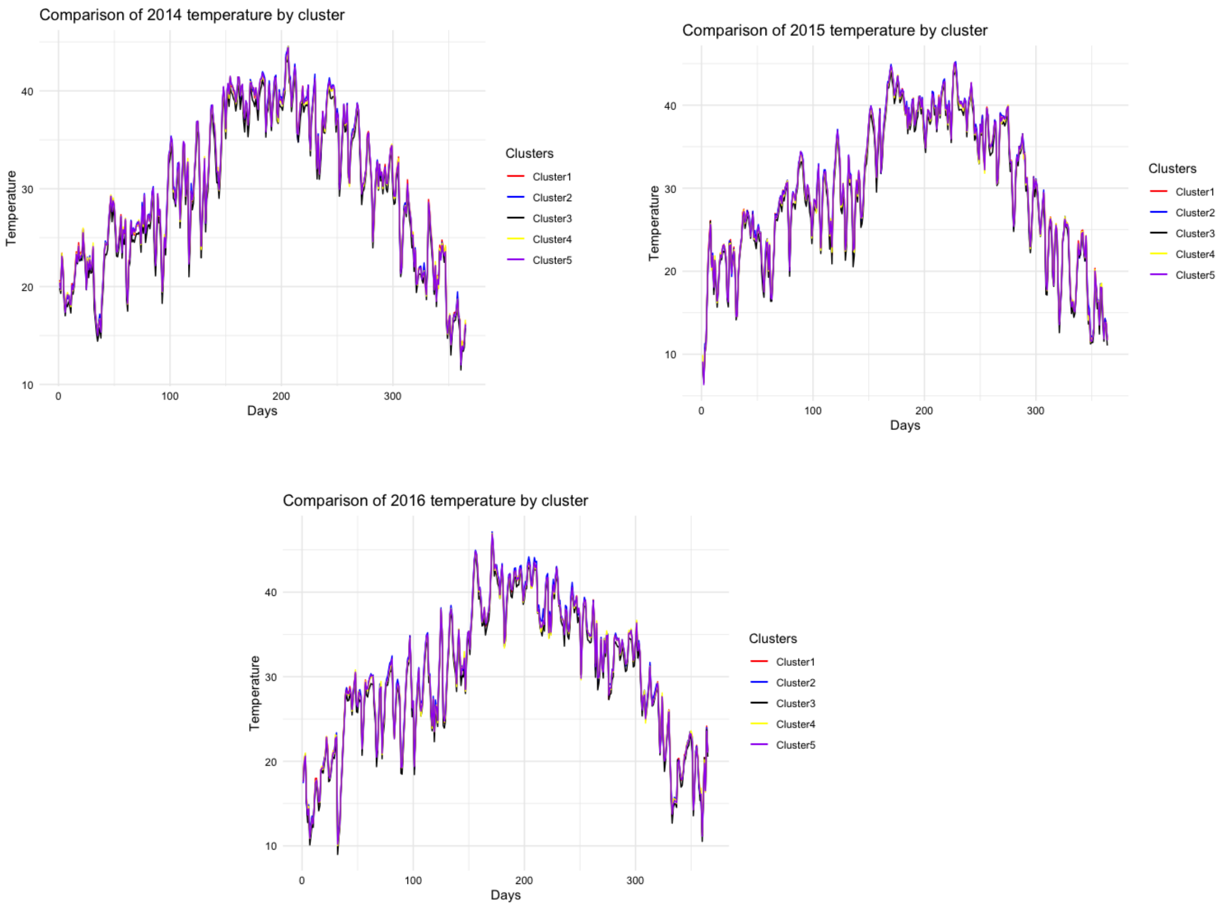

Figure 6: Daily temperature patterns for five clusters in 2014-2016. Each panel shows the mean temperature trajectory across the year for clusters identified from the corresponding year's data.

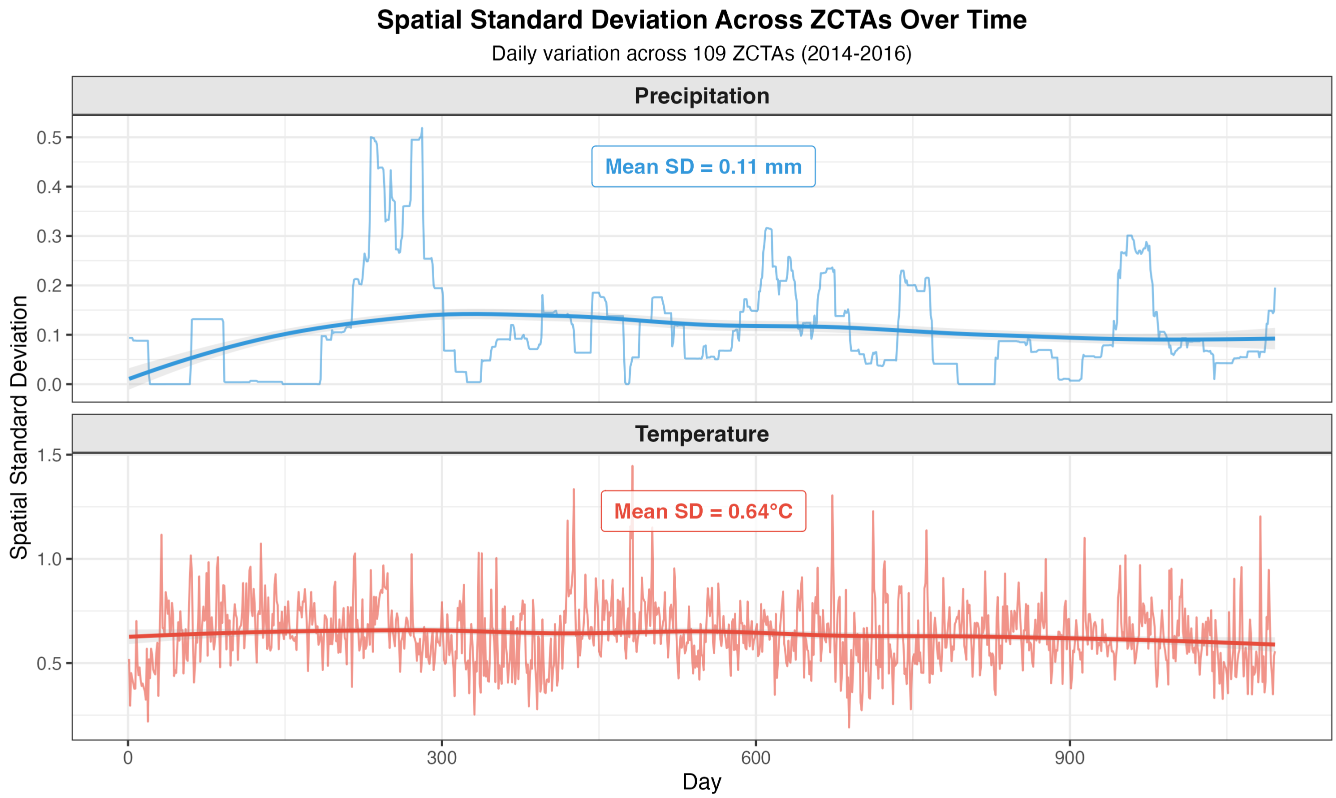

Figure 7: Spatial standard deviation of temperature and precipitation across ZCTAs over time. Time series showing daily spatial standard deviation (variation across 109 ZCTAs) for (Top) precipitation and (Bottom) temperature during the 2014-2016 study period. Lines show daily spatial SD values with LOESS smoothing (shaded areas indicate 95% confidence intervals). Temperature shows consistently low spatial variation (mean SD = 0.64°C, CV = 0.021), while precipitation shows episodic spatial heterogeneity corresponding to monsoon events (mean SD = 0.11 mm, CV = 0.30). The 14-fold difference in coefficient of variation confirms that spatial heterogeneity in precipitation is substantial relative to its magnitude, while temperature spatial variation is negligible.

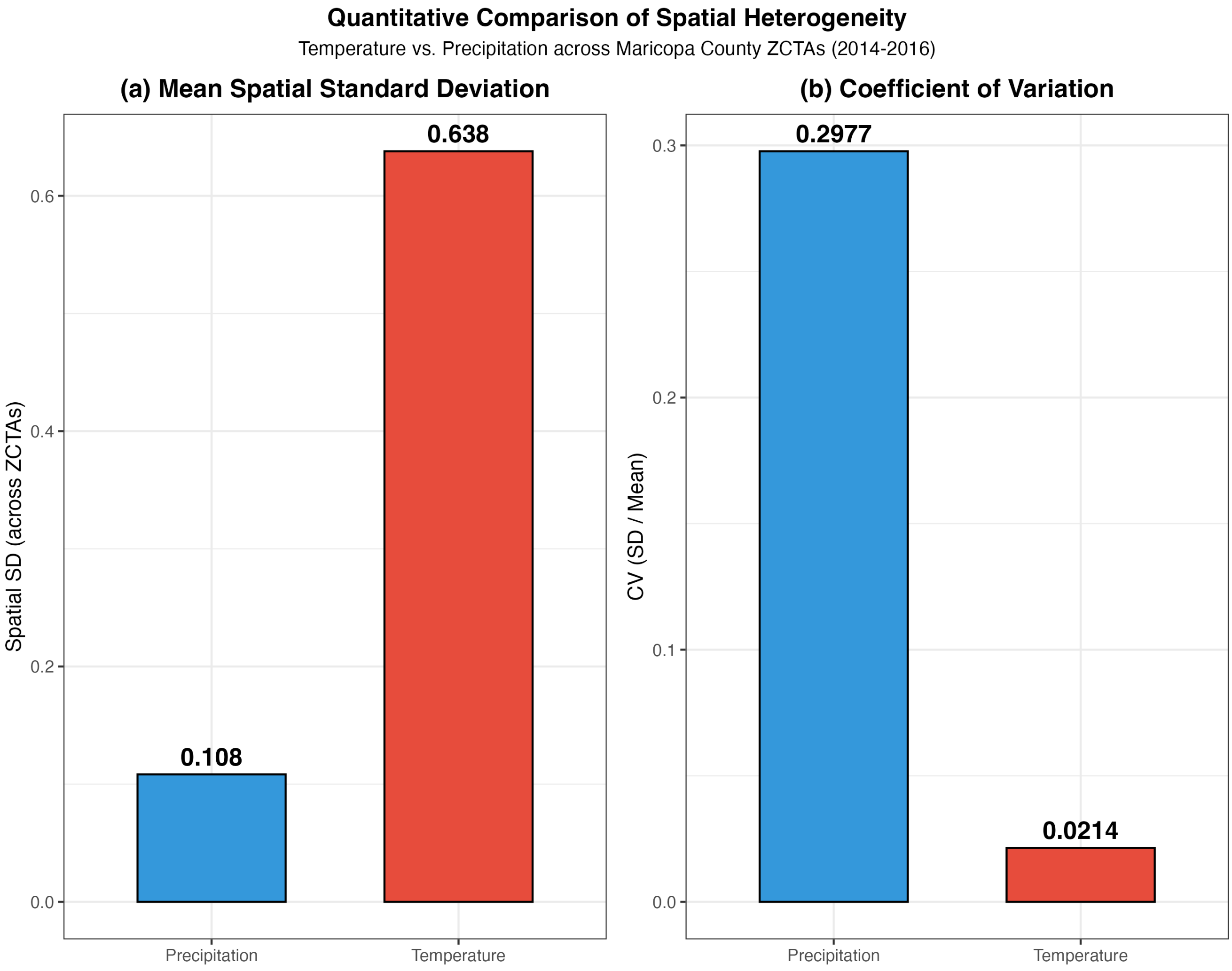

Figure 8: Quantitative comparison of spatial heterogeneity between temperature and precipitation. Bar plots comparing (a) mean spatial standard deviation (SD) across all days and ZCTAs, and (b) coefficient of variation (CV = SD/mean) for temperature and precipitation. While temperature has a larger absolute spatial SD (0.64°C vs. 0.11 mm), precipitation has a 14-fold larger coefficient of variation (0.30 vs. 0.021), indicating greater relative spatial heterogeneity. The CV is the appropriate metric for comparing variables with different units and magnitudes. Temperature spatial variation represents only 2.1% of the mean value, confirming negligible spatial heterogeneity, while precipitation spatial variation represents 30% of the mean, confirming substantial heterogeneity that justifies spatial clustering.

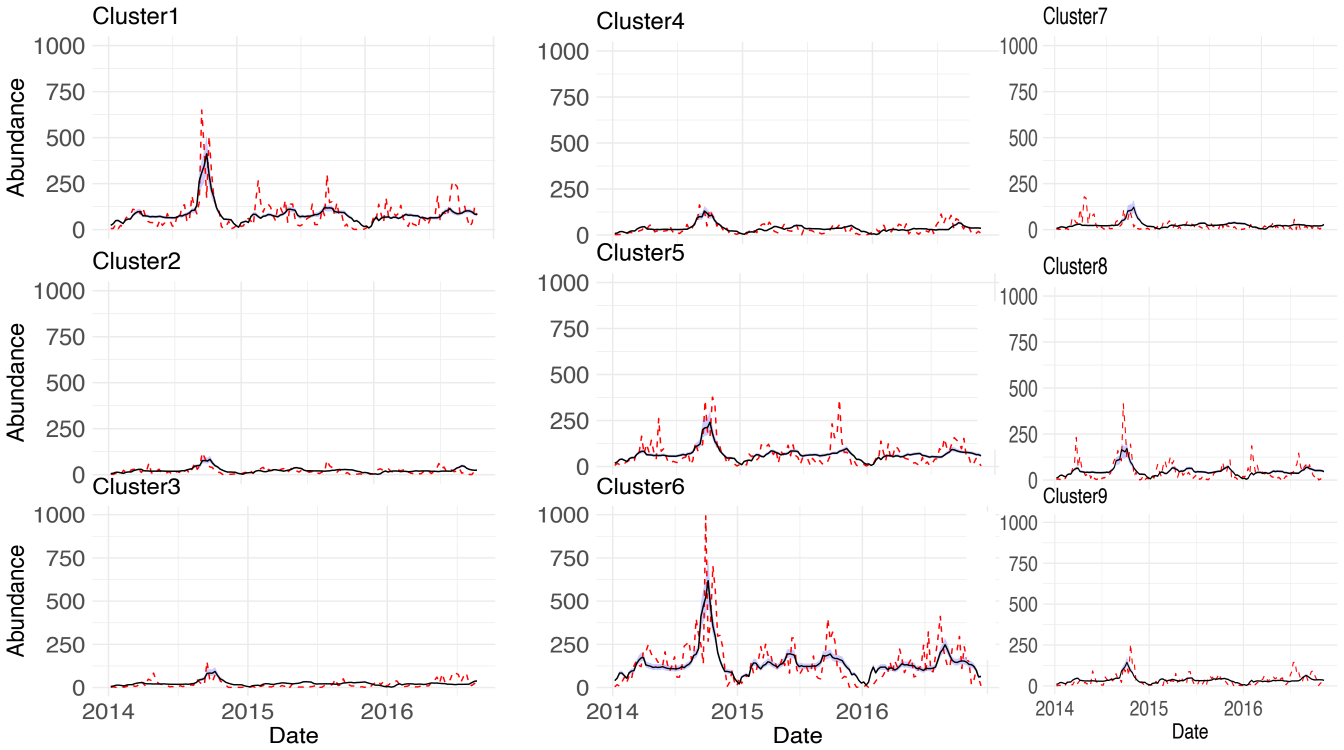

Figure 9: 9 cluster prediction. Shaded ribbons indicate 95% credible intervals from the county fit. Red dotted lines are the real data, while the black line is the model fit for within-sample fits.

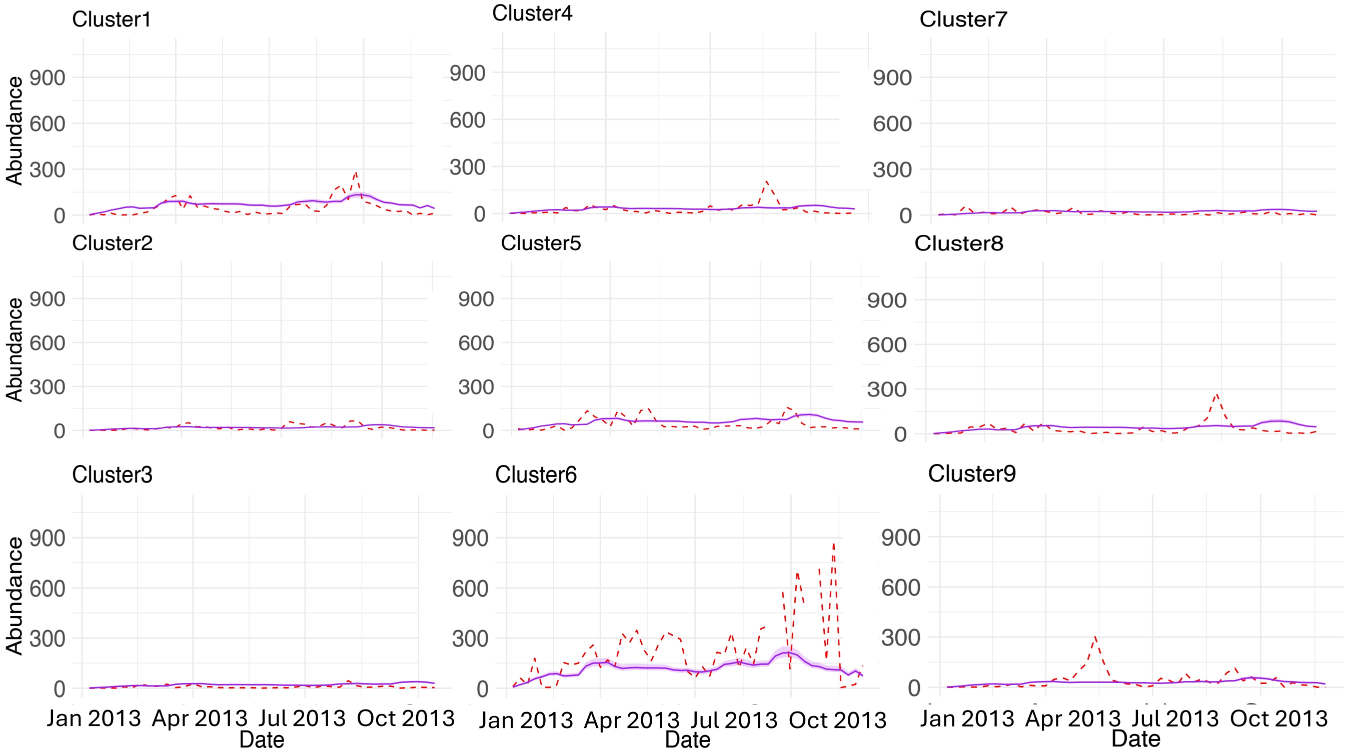

Figure 10: 9 cluster 2013 prediction. Shaded ribbons indicate 95% credible intervals from the county fit. Red dotted lines are the real data, while the orange line is for out-of-sample fits for 2013.

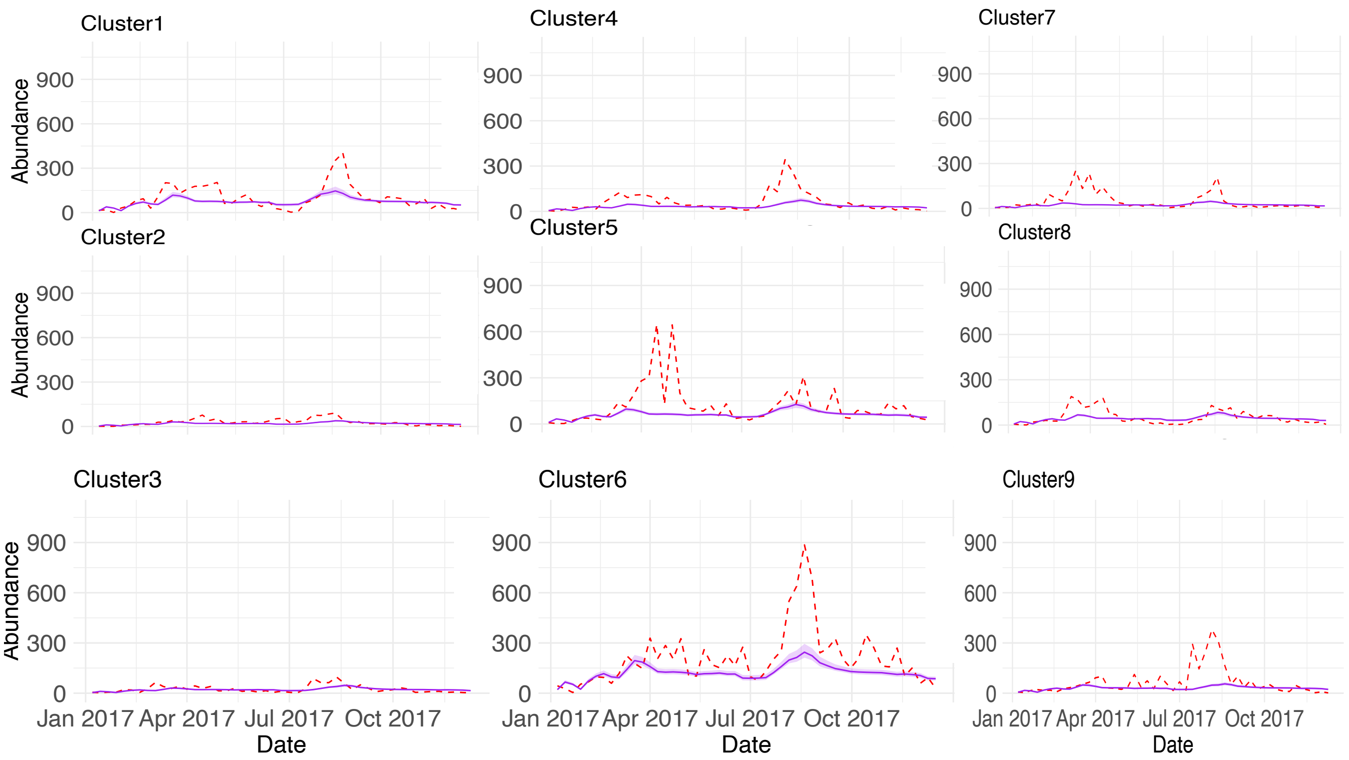

Figure 11: 9 cluster 2017 prediction. Shaded ribbons indicate 95% credible intervals from the county fit. Red dotted lines are the real data, while the orange line is for out-of-sample fits for 2017.

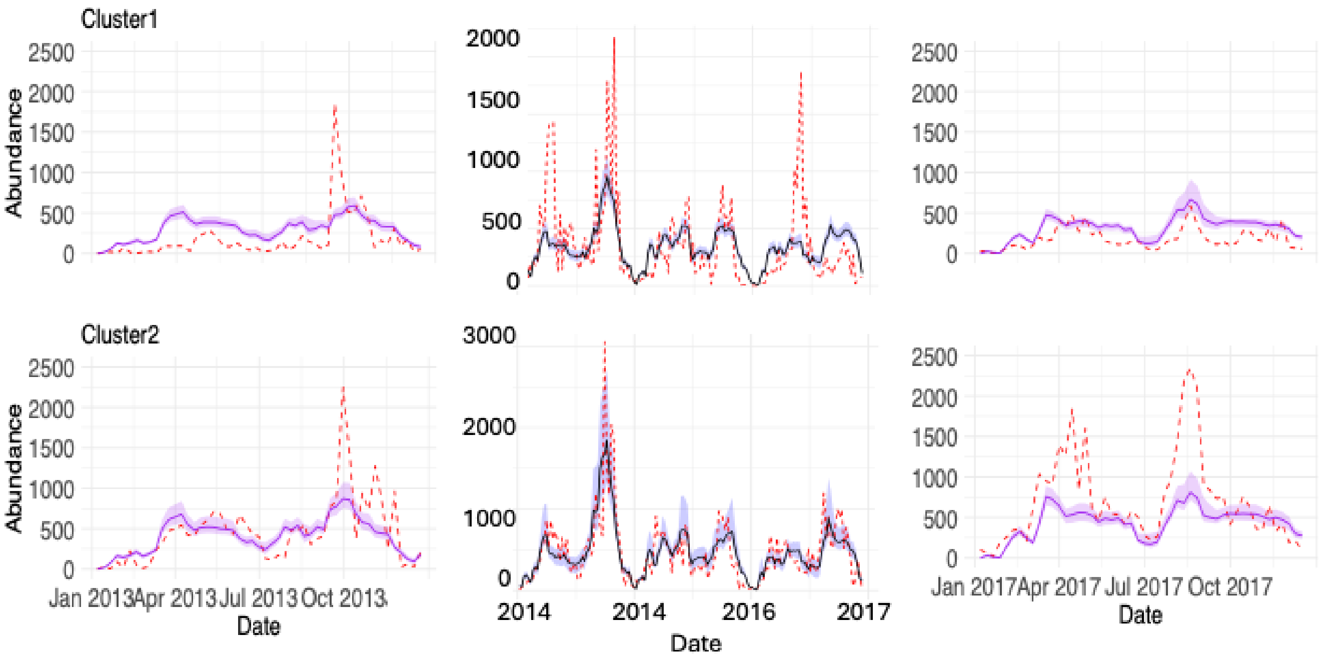

Figure 12: 2 cluster prediction. Shaded ribbons indicate 95% credible intervals from the county fit. Red dotted lines are the real data, while the black line is the model fit for within-sample fits, and the orange line is for out-of-sample fits.

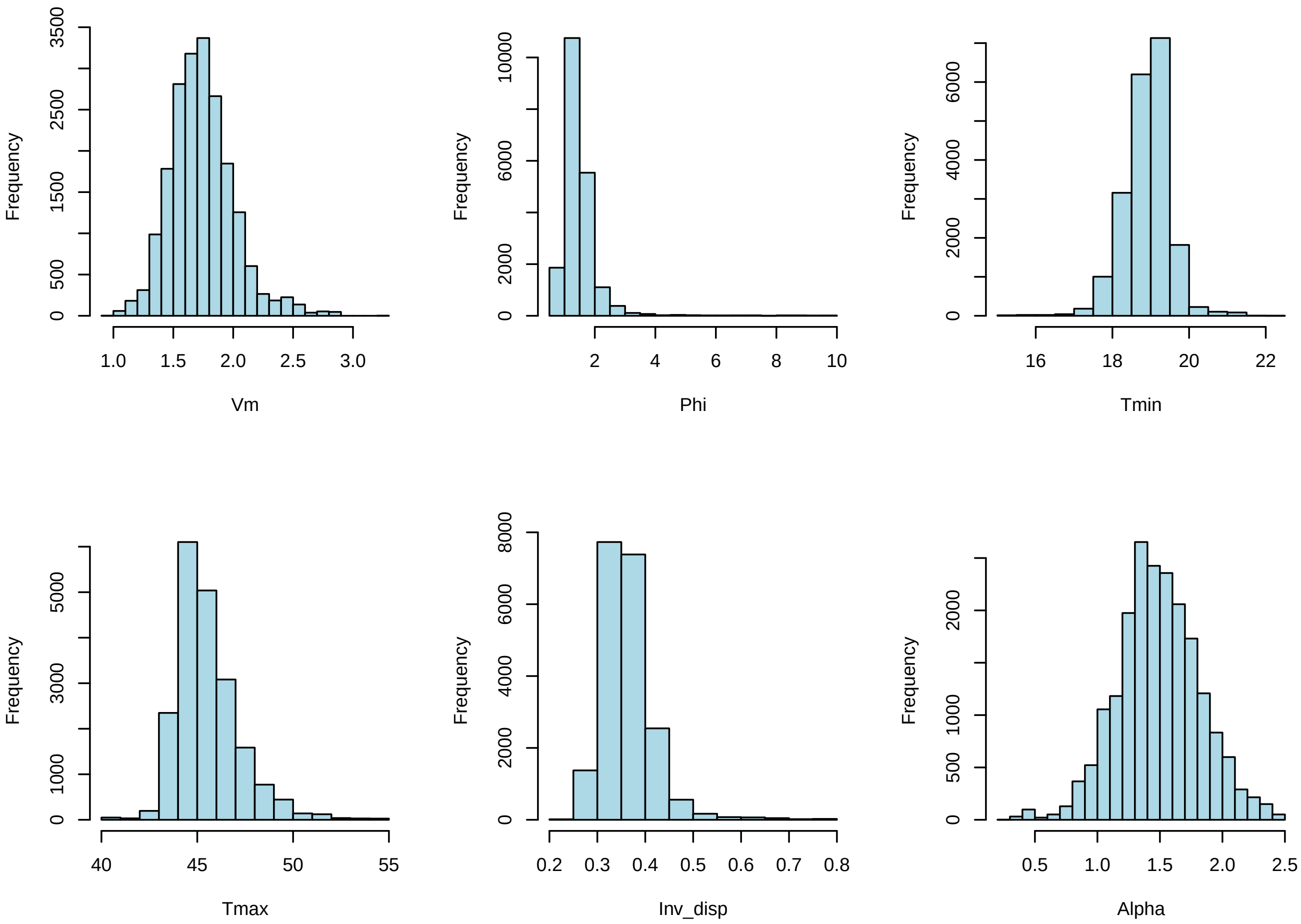

Figure 13: Histograms of marginal posterior draws of parameters in the county-level model-fitting without outlier ZTCA.

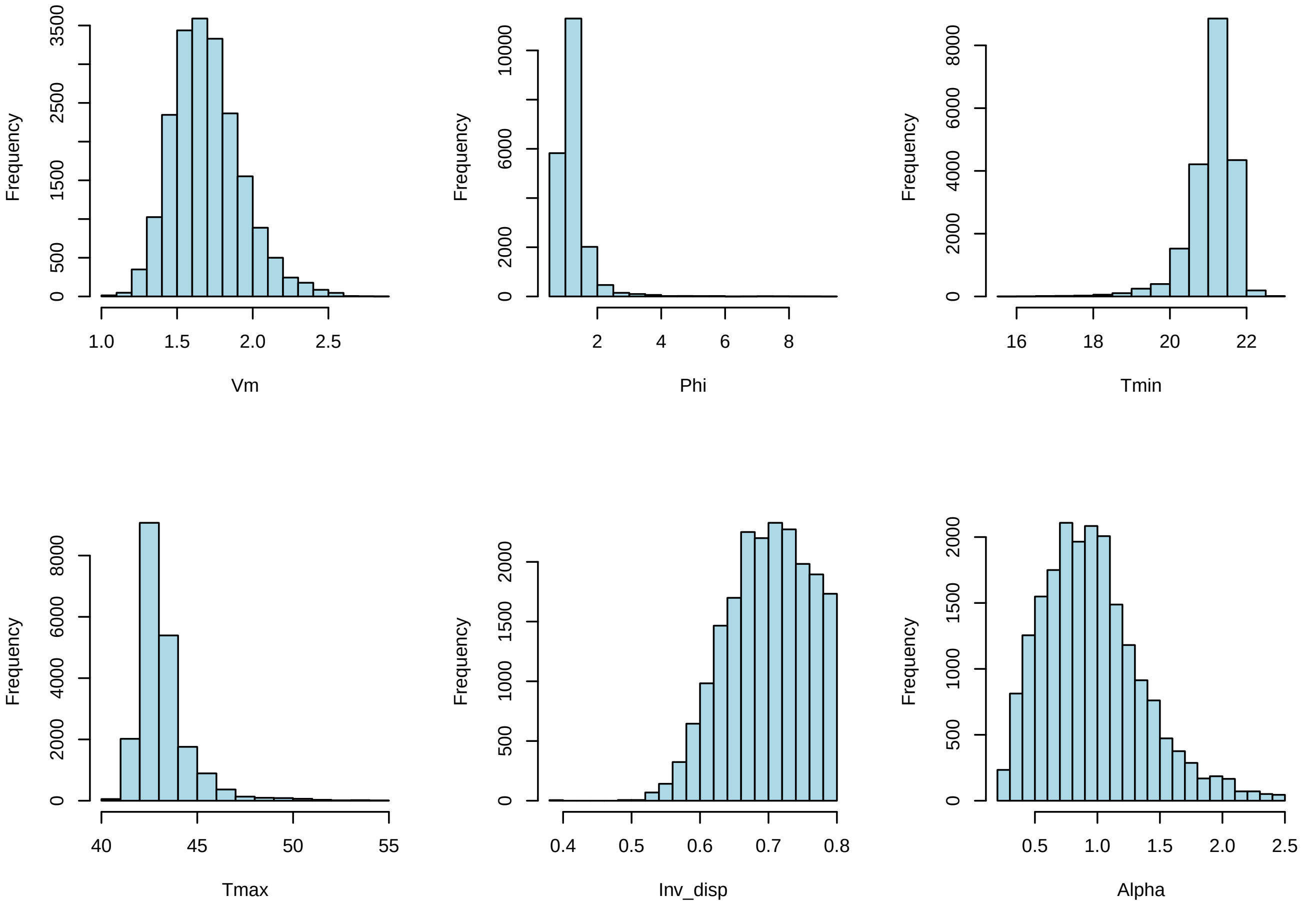

Figure 14: Histograms of marginal posterior draws of parameters for outlier ZTCA model fitting.

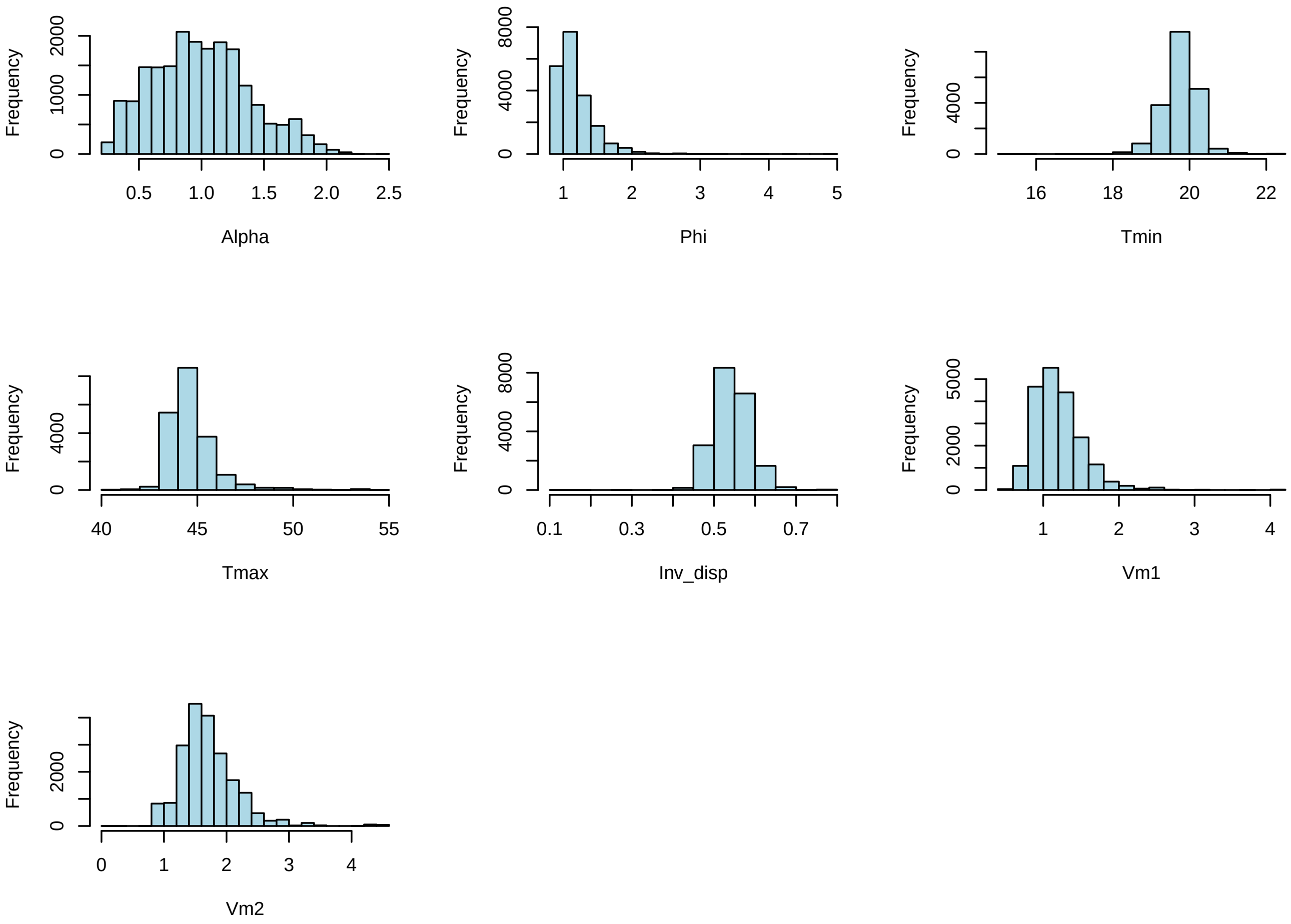

Figure 15: Histogram of marginal posterior draws of parameters for 2-cluster model fitting.

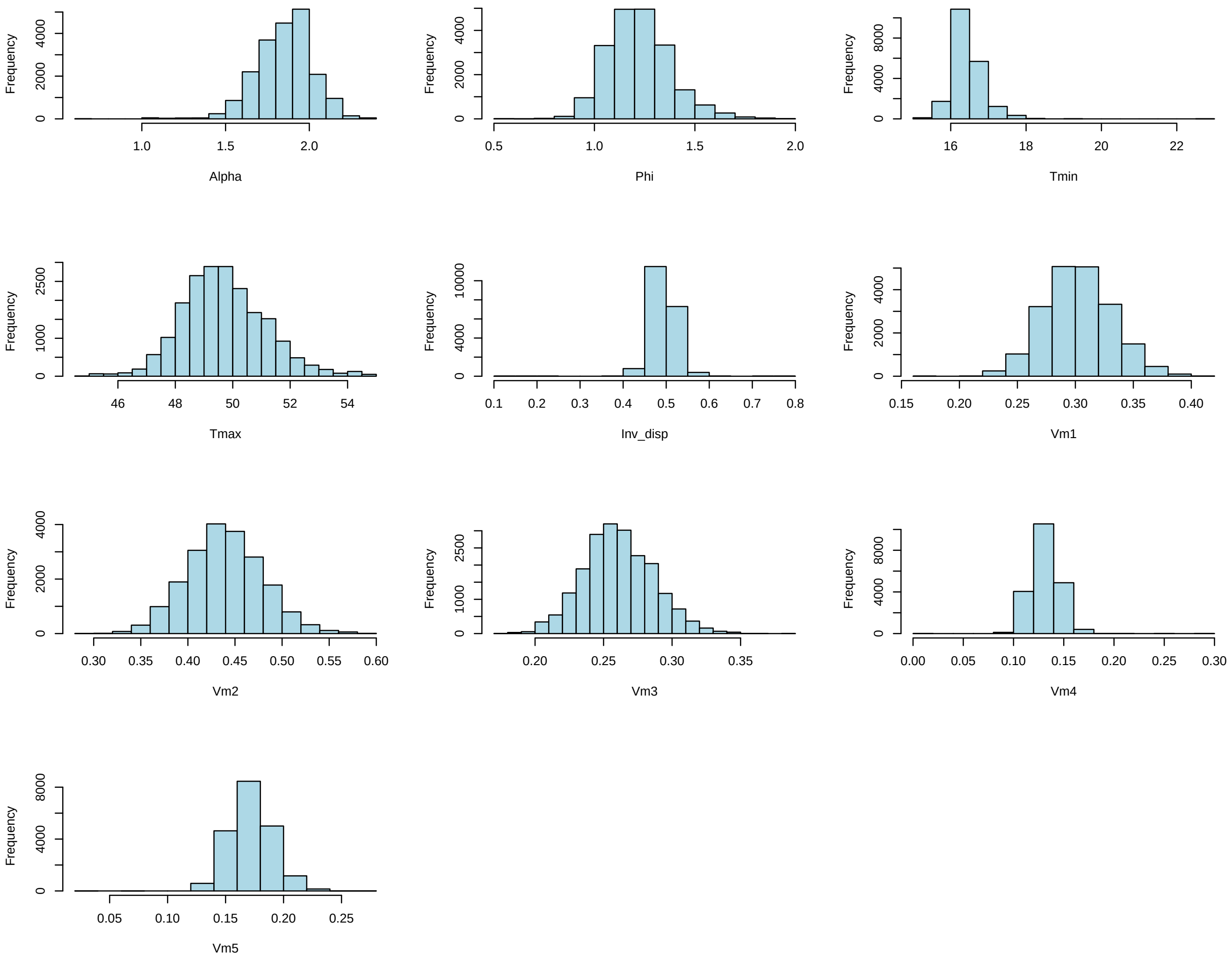

Figure 16: Histogram of marginal posterior draws of parameters for 5-cluster model fitting.

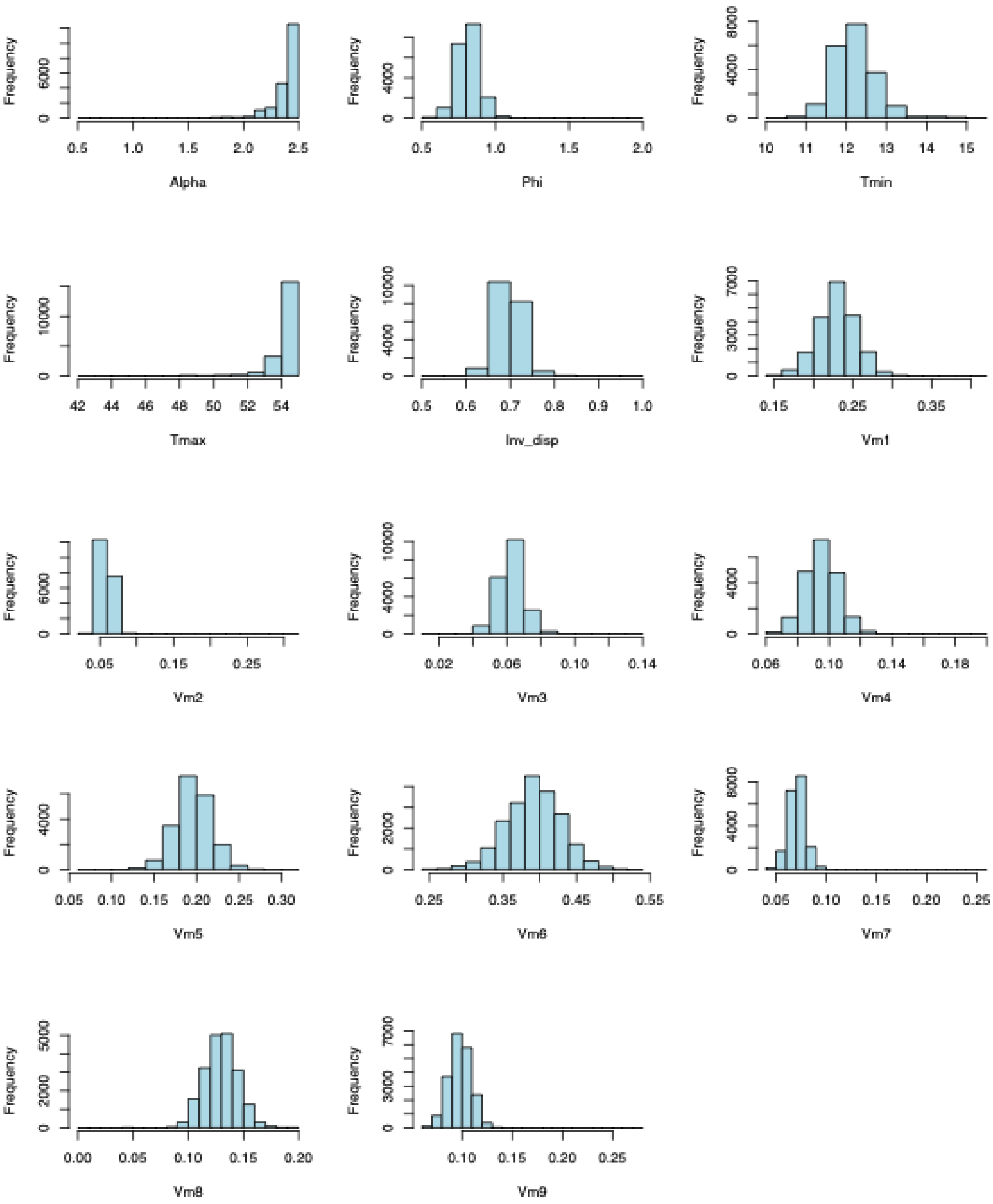

Figure 17: Histogram of marginal posterior draws of parameters for 9-cluster model fitting.

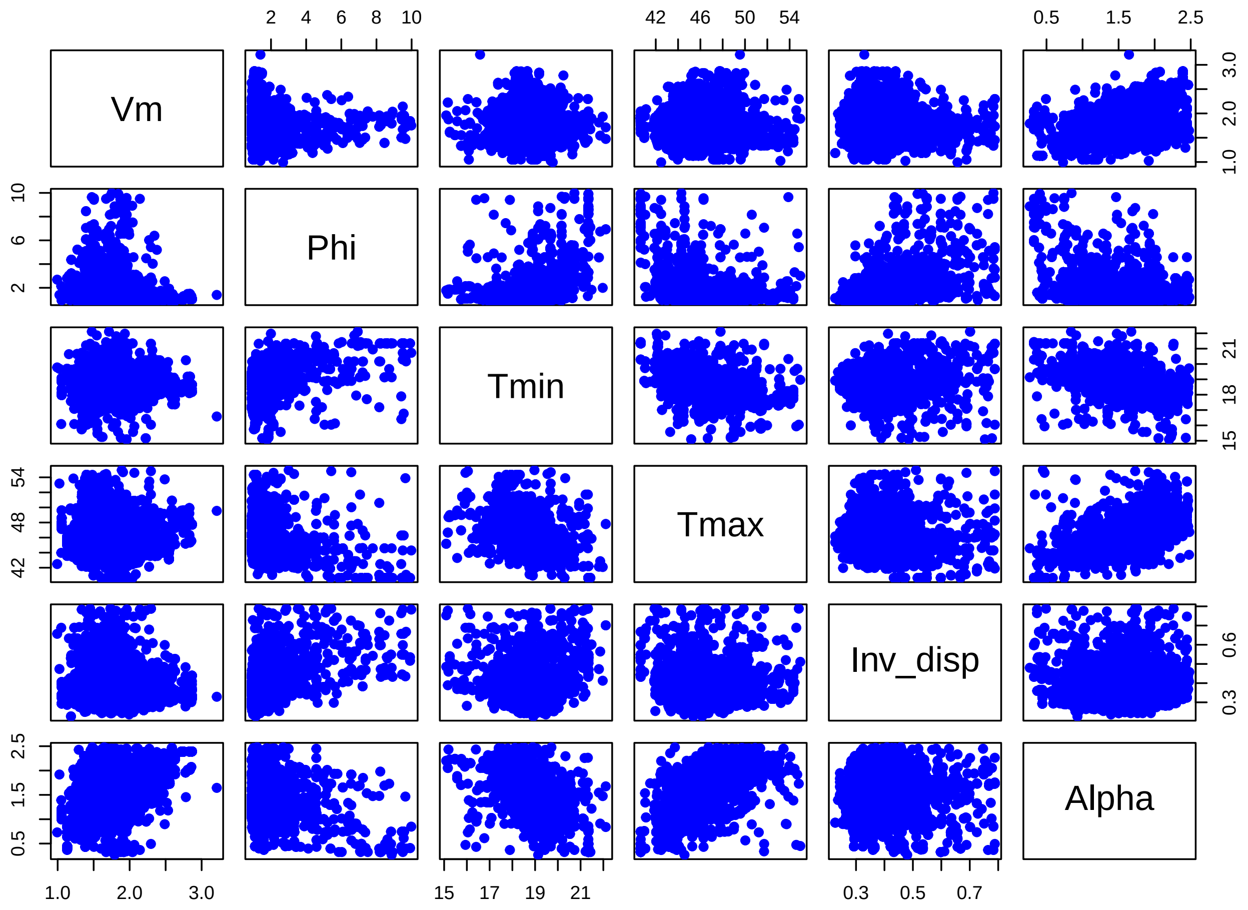

Figure 18: Pairs plot displaying joint posterior draws of parameters from the county-level model without outliers.

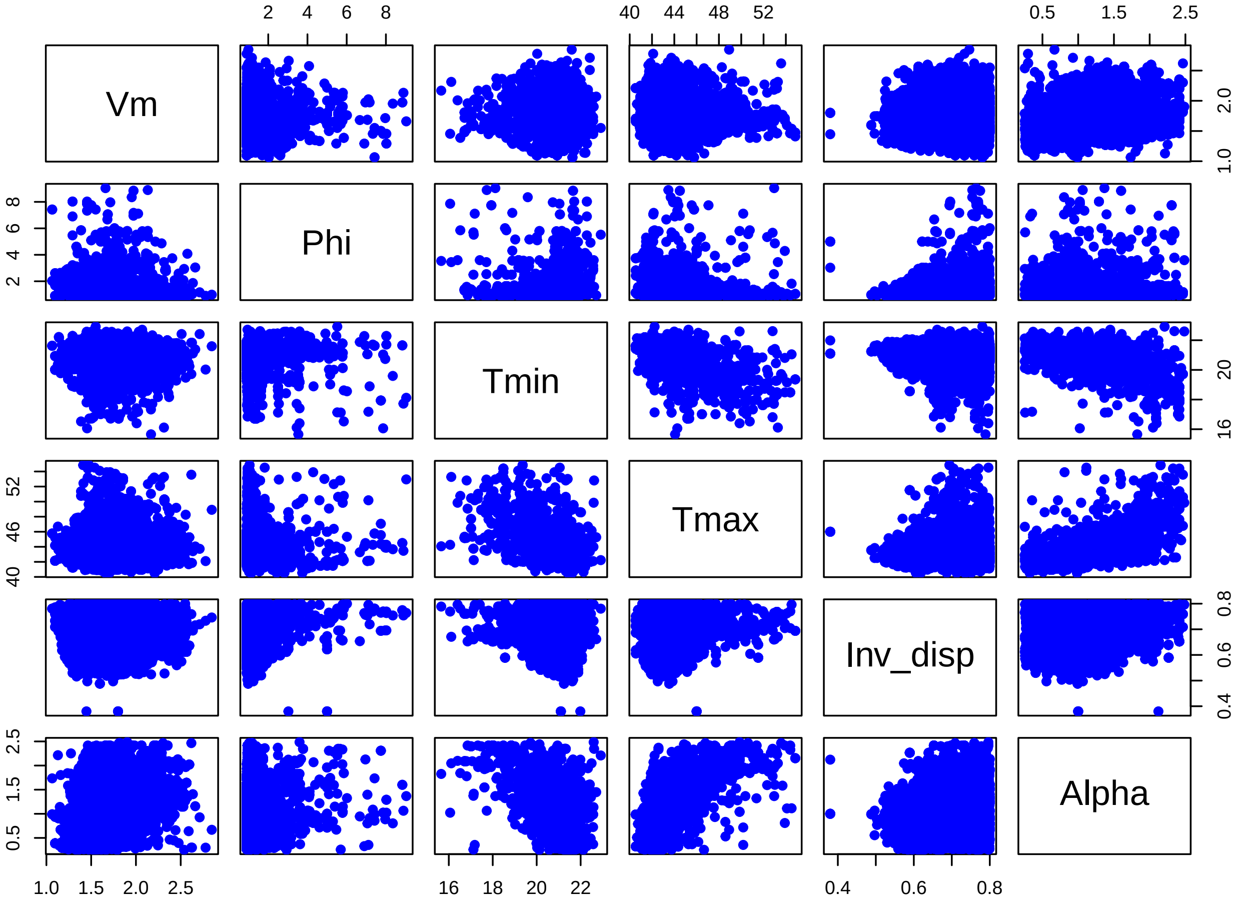

Figure 19: Pairs plot displaying joint posterior draws of parameters from the outlier ZCTA model.

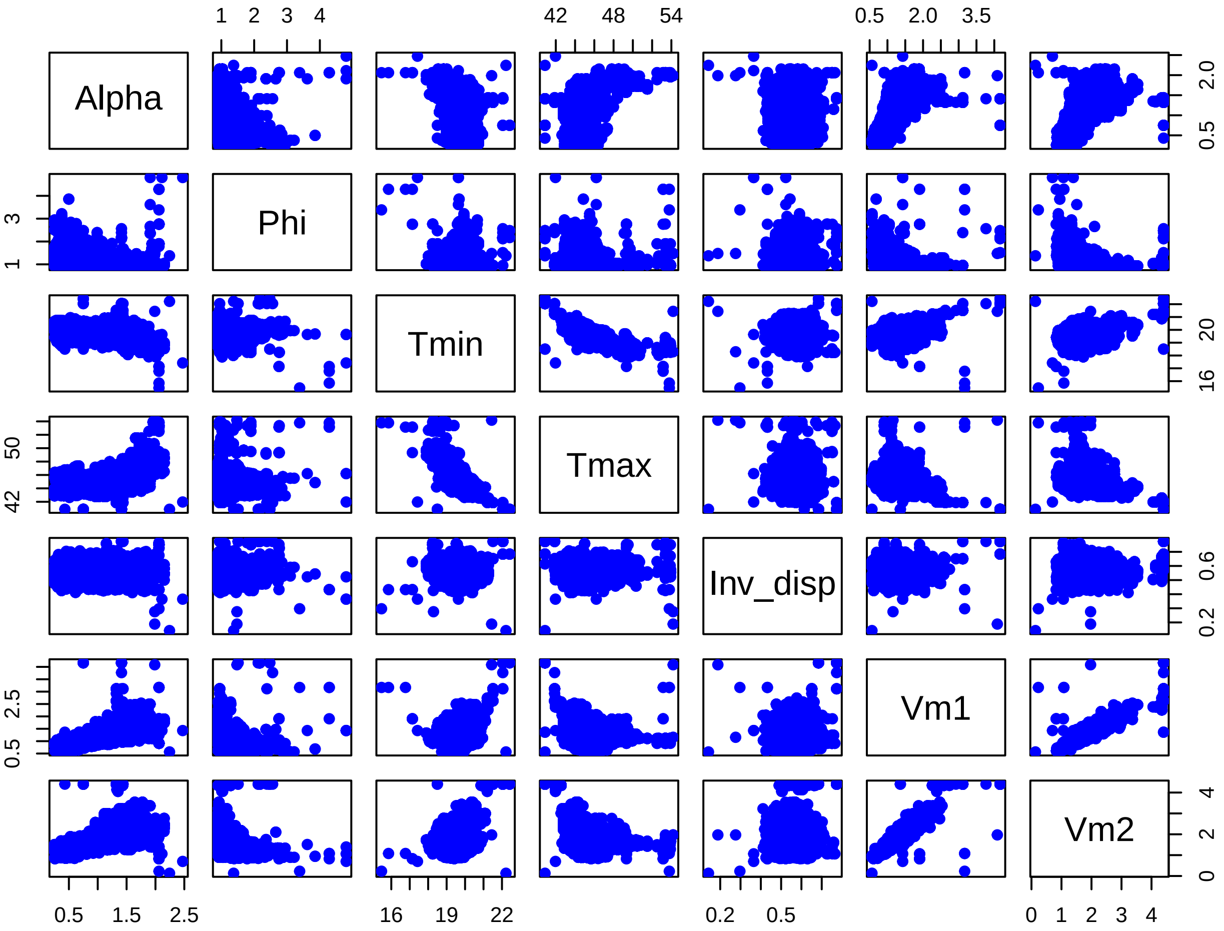

Figure 20: Pairs plot displaying joint posterior draws of parameters from 2 cluster model.

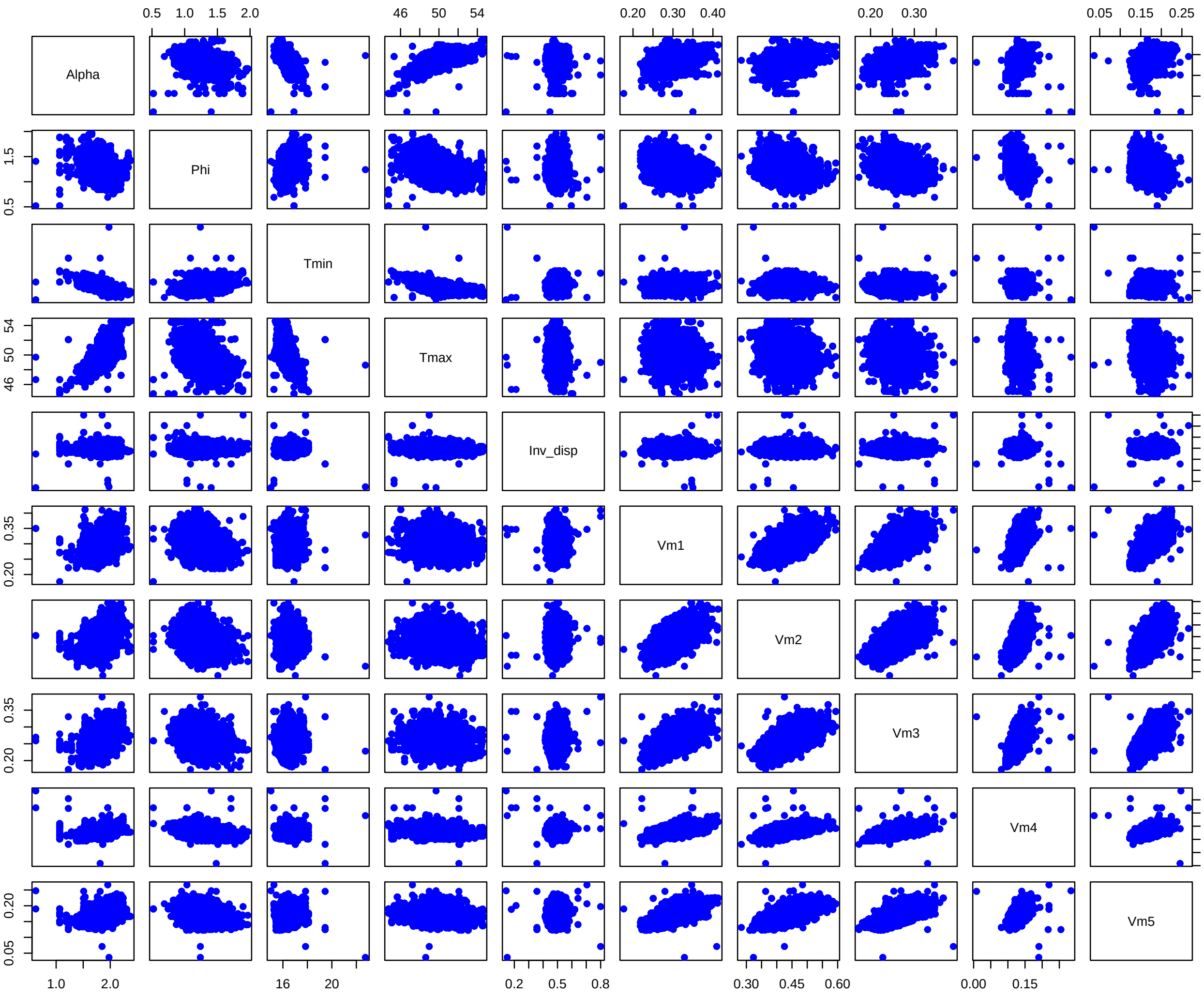

Figure 21: Pairs plot displaying joint posterior draws of parameters from 5 cluster model.

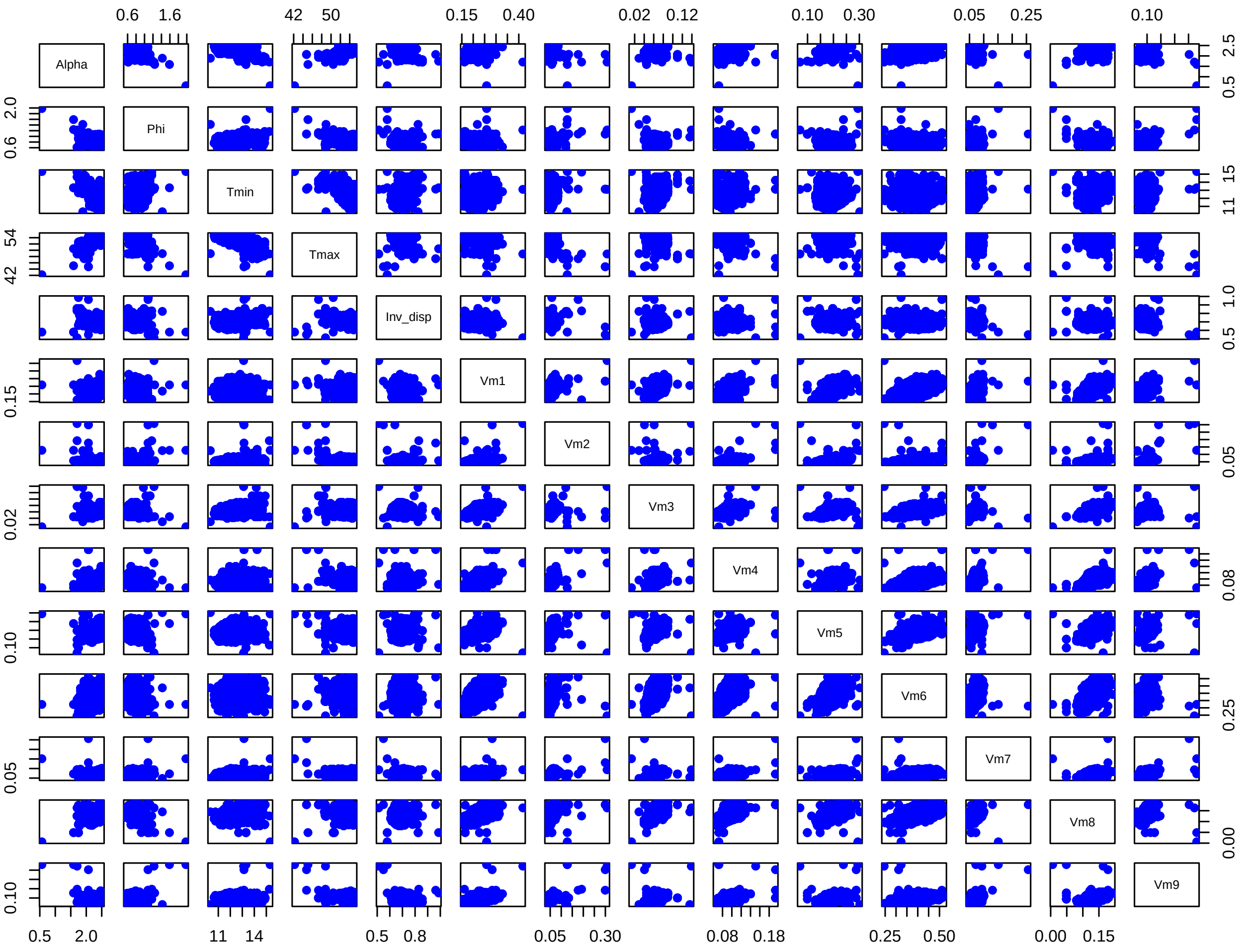

Figure 22: Pairs plot displaying joint posterior draws of parameters from 9 cluster model.

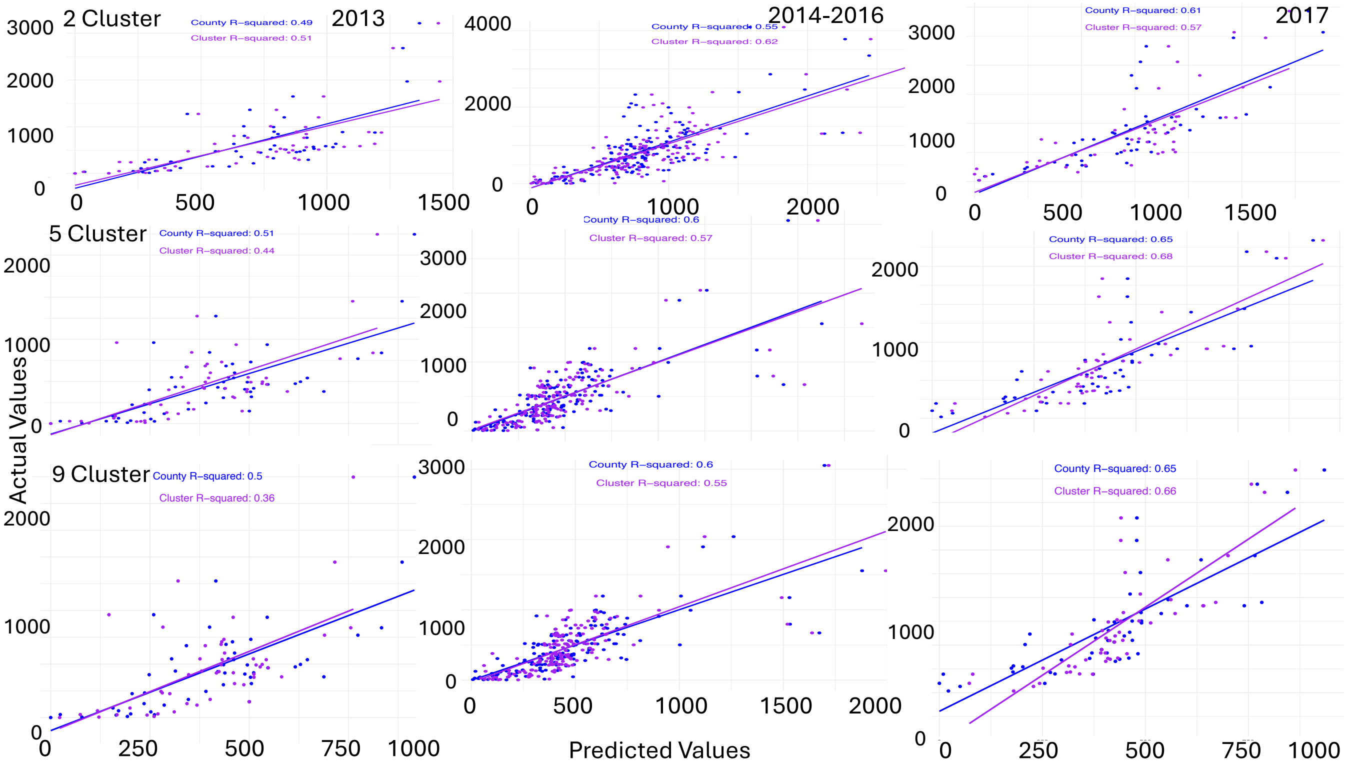

Figure 23: Predicted vs Actual for all clusters
